# Tidal inlet seafloor changes induced by recently built hard structures

**DOI:** 10.1371/journal.pone.0223240

**Published:** 2019-10-16

**Authors:** Carlotta Toso, Fantina Madricardo, Emanuela Molinaroli, Stefano Fogarin, Aleksandra Kruss, Antonio Petrizzo, Nicola Marco Pizzeghello, Luigi Sinapi, Fabio Trincardi

**Affiliations:** 1 Istituto di Scienze Marine-Consiglio Nazionale delle Ricerche, Arsenale - Tesa 104, Castello 2737/F, 30122 Venezia, Italy; 2 Department of Environmental Sciences, Informatics and Statistics (DAIS), Università Ca’ Foscari Venezia, Campus Scientifico, Via Torino 155, Mestre, VE, Italy; 3 Istituto Idrografico della Marina, Passo all’Osservatorio 4, Genova 16134, Italy; 4 Dipartimento Scienze del Sistema Terra e Tecnologie per l’Ambiente, Piazzale Aldo Moro 7, Roma, Italy; Universidade de Aveiro, PORTUGAL

## Abstract

Tidal inlets are extremely dynamic environments that are often strongly modified by anthropogenic intervention. In this study, we describe the rapid evolution of a highly human-impacted tidal inlet, studied through repeated high-resolution multibeam surveys and geomorphometric analysis. We document the rapid change induced by new hard coastal structures built to protect the historical city of Venice (Italy). A new breakwater erected between 2011 and 2013 induced the formation of large scour holes with the consequent erosion of about 170 · 10^3^ ± 15.6% m^3^ of sediment until 2016. The construction of a new island in the middle of the inlet and the restriction of the inlet channel caused a general change of the inlet sedimentary regime from depositional to erosive with a net sediment loss of about 612 · 10^3^ ± 42.7% m^3^, a reduction of the dune field area by more than 50% in about five years, and a coarsening in the sediment distribution. Our results give new insight on the tidal inlet resilience to changes, distinguishing two different phases in its recent evolution: (i) a very rapid response (from 2011 to 2013) of the seafloor morphology with scour-hole erosion at the new breakwater tips at a rate of about 45⋅10^3^ m^3^/year and the disappearing of dune fields at a rate of 104⋅10^3^ m^2^/year; and (ii) a general slowdown of the erosive processes from 2013 to 2016. Nevertheless, the erosion continues at the breakwater, though at a reduced rate, possibly representing a threat to the hard structure. In view of global mean sea level rise and consequent proliferation of hard structures along the coast all over the world, the combined use of very high resolution multibeam surveys and repeatable geomorphometric analysis proposed in this study will be crucial for the monitoring and future management of coastal environments.

## Introduction

Coastal systems are among the most productive and yet most threatened ecosystems in the world [1]. In addition, it is estimated that 40% of the world population lives within the coastal zone, with a further increase occurring during the touristic season [2]. On the whole planet, coastlines extend for more than 1.6 million km and coastal ecosystems can be found in 123 countries [3]. Among coastal systems, coastal lagoons occupy 13% of the global coastal surface [4]. Coastal lagoons are connected to the open sea through tidal inlets that allow water, sediment and nutrients exchange [5]. Nowadays tidal inlets are increasingly influenced by human intervention and activities such as the construction of hard structures [6], [7], fishing, seaborne transport and recreational activities ([8]), and dredging for navigation purposes ([9]; [10]). However, the long-term response of the inlet seafloor morphology to human interventions and activities is still poorly understood ([10]). Only recently technological advances in the multibeam echosounder instruments allow high resolution mapping in very shallow water and provide new insight on seafloor transport processes related to these activities ([5]; [11]). Moreover, in view of the global mean sea level rise and coastal wetland vulnerability ([12]; [13]), the physical impact of new protection structures on the seafloor becomes particularly relevant.

**Fig 1:**
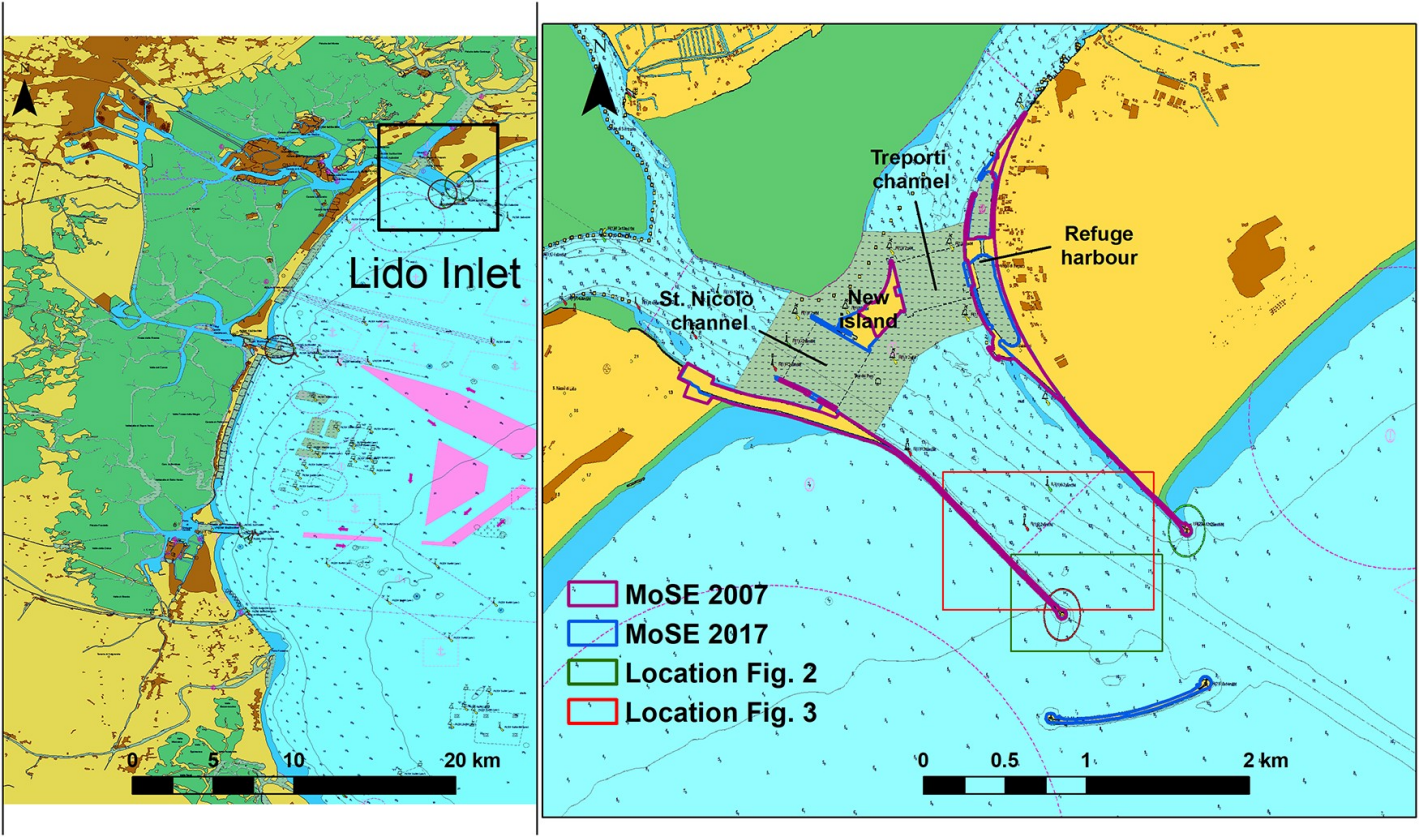
Study area. Left: Venice Lagoon, Italy; right: images of Lido Inlet. The pink polygons represent the MoSE structures in 2007 after the construction of part of the island at the lagoon side of the inlet and the structures at the inlet channel sides; the blue polygons depict the MoSE structures in 2017 after the construction of the breakwater at the seaside. The green and red rectangles represent the locations of Figs 2 and 3, respectively. Reprinted from Nautical Chart 226 and 222 under a CC BY license, with permission from Italian Hydrographic Institute, original copyright 2016. The graphical representation of the figure is compliant with the official rules used for the nautical charts defined by the International Hydrographic Organization (the french version of the rules integrated with the english meaning can be downloaded at https://www.iho.int/iho_pubs/standard/S-4/INT1_FR_Ed7_2019.pdf). Light blue-green: water areas at different depths; Grey: water area with ongoing works; Light pink: water area regulated for navigation; Yellow-brown: land area.

Since 2002, at the inlets of the Venice Lagoon (Italy) (Fig 1) large mobile barriers have been under construction to protect the historical city of Venice (MoSE Project, [14]). MoSE is a system of mobile barriers positioned within each of the three inlets of the Lagoon of Venice ([15]), built to limit the flooding of the city of Venice ([16]; [17]; [18]). These barriers lie on the seafloor during normal conditions, being raised when there are floods with water levels higher than 110 cm above the vertical datum of Punta Salute (1897) (ZMPS—Zero Mareografico Punta Salute) ([19]; [17]), used locally as reference for tidal measurements.

The processes induced by the construction of coastal hard structures are well studied in the scientific literature (e.g. [9], and references therein for a review). These studies are mainly based on laboratory experiments (e.g. [20]) and/or numerical models (e.g. [21]).

Shallow-water Multi-Beam Echo Souder System (MBES) has been widely used in mapping seabed morphology and composition (e.g. [22]; [23]; [24]; [25]; [26]; [27]), pipeline routes ([28]; [29]), coral reefs ([30]; [31]), wrecks ([32]; [33]), mines and the extent of their burial ([34]; [35]). MBES has recently been used for monitoring the seafloor scouring close to offshore windfarm foundations (e.g. [36] and reference therein) and bridge piers in rivers (e.g. [37]; [38]). Recent applications have shown the potential of MBES to map seafloor features and habitats at very high resolution (e.g. [39]; [40]; [41]).

In the last decade, geomorphometric methods to characterize quantitatively terrestrial landscapes (e.g. terrain attributes, feature extraction, automated classification) became well established and have been extensively applied to the marine environment ([42],[43]). The most recent studies based on high resolution repeated multibeam surveys focused on assessment of change in patterns of habitat distribution or on natural spatio-temporal morphological evolution (see for example [44]; [45]; [46]; [47]; [48]; [49]; [50]; etc.). However, as far as we know, the combination of high resolution data from MBES surveys and geomorphometric and sedimentological analysis has not been used to assess quantitatively and monitor the seafloor changes over time induced by newly built hard defence structures and more generally by coastal infrastructures.

This study investigated the mid-term effects of the construction of hard structures related to the MoSE project, employing a set of three repeated MBES bathymetric surveys, carried out over a period of five years. The aim was to assess the inlet evolution through the monitoring of the seabed sedimentary regime of the inlet system. To achieve this aim, we classified the inlet seafloor morphologies with a repeatable semi-automatic geomorphometric analysis of the digital elevation model of each survey ([43]). Moreover, considering the global increase of hard coastal defence structures to protect the shoreline from global mean sea level rise ([51]; [52]), our methodology can be applied to other coastal areas around the world for the future management of new coastal defence structures.

## Study area

The lagoon of Venice (Fig 1) is located at the northern tip of the Adriatic Sea, along the eastern coast of Italy (45°N, 12°E). This is the largest coastal lagoon in the Mediterranean area ([53]), covering a surface of 550 km^2^ (it stretches for 50 km along the coastline, with a mean width of 15 km and an average depth of 1.5 m) ([54]). The tide is semidiurnal with an average range of 55 cm increasing to 110 cm during spring tides ([55]) and the residence time varies between 24 hours close to the inlets ([56]; [57]) and 30 days in the internal lagoon ([58]; [54]). The lagoon today exchanges water and sediment with the Adriatic Sea through three wide inlets ([59]): Lido, Malamocco and Chioggia inlet from north to south (Fig 1 left). The total exchange of water with the sea is about 350 ⋅ 10^6^ m^3^ during spring tides and 175 ⋅ 10^6^ m^3^ during neap tides ([60]).

The Lido inlet (45°25′18″N,12°26′0″E—Fig 1) is the northernmost inlet and is crossed by cruise ship and ferry traffic. The tidal prism at Lido inlet is about 145 ⋅ 10^6^ m^3^ ([61]). Like the rest of the Lagoon, the Lido inlet is exposed to two major wind events; the scirocco, an autumnal/spring wind that blows from south-east, and the bora, that prevails in winter and blows from north-east ([55]). Until 1882, there were three channels in this area ([62]), but between 1882 and 1910 the building of the jetties still present today merged these three channels into one and fixed the inlet position ([63]) (Fig 1 right). In the last 15 years, the inlet configuration has changed again, due to the construction of the MoSE structures (Fig 1 right). The most relevant intervention were the construction of the island (from 2004 to 2008) and of the breakwater (from 2010 to the end of 2012) (Table 1).

**Table 1:** Latest Interventions at Lido inlet. In Fig 1 are shown the main locations.

Year	Intervention
2004	The construction of the refuge harbour begins.
	The reinforcement of the soutern jetty begins: construction of a reef parallel to the existing one.
	The construction of the new island begins.
	St. Nicolò channel: the reinforcement of the seafloor with boulders and stones where the mobile gates are going to be positioned begins.
2006	The construction of the refuge harbour continues, and the navigation basin realization begins.
	Treporti channel: protection of the seafloor with bouders and stones.
	St. Nicolò channel: the construction of the southern abutment of the mobile gates begins.
	St. Nicolò channel: the excavation of the MoSE trench begins.
2007	The core of the new island is completed. The abutment of the mobile gates is still under construction.
2008	The construction of the new intermediate island is completed.
	The construction of the shelter is completed. Also the navigation basin is almost finished.
	The production of the caissoins begins.
	Treporti channel: construction of the eastern abutment of the mobile gates.
	The reinforcement of the soutern jetty is almost completed.
2010	The seafloor protection is completed.
	The excavation of the MoSE trench is completed.
	The construction of the breakwater outside the inlet begins.
2012	The breakwater is almost completed.
	Positioning of the caissoins in the Treporti channel.
2013	All the construction interventions at the inlet are completed.
	The first four mobile gates are installed in the Treporti channel.
	Positioning of the caissoins in the St. Nicolò channel.
	First trial of the functioning of the barrier in Treporti channel.
2014	All the mobile gates of Treporti channel are installed.
	All the caissoins in St. Nicolò channel are positioned.

## Materials and methods

### Multibeam data acquisition

Bathymetry and backscatter data have been acquired in a time span of six years, from 2011 to 2016, for a total of three datasets. Dataset 1 (2011) was acquired by the Italian Hydrographic Institute (I.I.M.) of the Italian Navy during a nautical chart updating campaign. It took two different campaigns to cover the whole area of Lido inlet (the first from 7^th^ to 15^th^ September 2011 and the second from 14^th^ September to 11^th^ October 2011). The dataset 2 (2013) was acquired by the researchers of the Italian Research Council (CNR-ISMAR) in June 2013. The dataset 3 (2016) was acquired in April and May 2016, both from the I.I.M. and CNR-ISMAR personnel, during two different surveys. The technical characteristics of all the surveys are summarised in Table 2. Despite some technical differences in the instruments adopted during the successive surveys and in the accuracy of the positioning systems, the three surveys can be reliably compared to support quantitative assessments on the modifications of the sea floor in the study area.

**Table 2:** Surveys setup.

	Dataset 1 (2011)—first survey	Dataset 1 (2011)—second survey	Dataset 2 (2013)	Dataset 3 (2016)	
**Investigator**	I.I.M	I.I.M.	CNR-ISMAR	CNR-ISMAR	I.I.M.
**MBES**	Kongsberg Simrad EM 3002 MBES	Kongsberg Simrad EM 3002 MBES	Kongsberg EM2040 Compact dual-head	Kongsberg 2040 Compact single-head dual swath MBES	Kongsberg EM2040 Compact dual-head MBES
**Vessel**	7-m long	7-m long	10-m long	10-m long	7-m long
**Frequency**	300 kHz	300 kHz	360 kHz	320 kHz	360 kHz
**Overlap**	30%	30%	30%	30%	30%
**Mean Speed**	6.3 kn	6.3 kn	6 kn	6 kn	6 kn
**Acquisition Software**	Kongsberg Seafloor Information System (SIS)	Kongsberg Seafloor Information System (SIS)	Kongsberg Seafloor Information System (SIS)	Kongsberg Seafloor Information System (SIS)	Kongsberg Seafloor Information System (SIS)
**Positioning System**	Kongsberg Seatex Seapath 300 positioning system, with NRTK correction	Differential Global Positioning System (DGPS) Kongsberg Seatex Seapath 300 with OMNISTAR Land corrections	Seapath 300 system supplied by a Fugro HP DGPS	Seapath 300 system supplied by a Fugro HP DGPS	Seapath 330 with a Fugro HP DGNSS corrections
**Motion Sensor**	Kongsberg Seatex MRU 5	Kongsberg Seatex MRU 5	Kongsberg Seatex MRU 5	Kongsberg Seatex MRU 5	Kongsberg Seatex MRU 5
**Sound Velocity Data Collection**	Idronaut Ocean Seven 316 multiparameter probe and a Valeport mini SVS sensor	Idronaut Ocean Seven 316 multiparameter probe and a Valeport mini SVS sensor	Valeport mini SVS sensor and AML oceanographic Smart-X sound velocity profiler	Valeport mini SVS sensor and AML oceanographic Smart-X sound velocity profiler	Idronaut Ocean Seven 316 multiparameter probe and a Valeport mini SVS sensor

The processing of data was the same for all the three datasets: the software CARIS Hydrographic and Side Scan Information Processing System ([64]) was used to take into account sound velocity variations, tides, and basic quality control of bathymetric data. All corrections were referred to the ZMPS. Backscatter data were processed with the Fledermaus Geocoder Toolbox (FMGT) software. Mosaics and terrain digital models, with a 0.5 m resolution, were created using the software ArcGIS v 10.2 ([65]).

### Sediment sampling

A total of 20 sediment samples were collected with a Van Veen grab in May 2016 and their position was strategically selected based on the main morphological features (Fig 5), according to the 2016 bathymetric map. In this operation a Seapath 300 system supplied by a Fugro HP DGPS was used. The samples were all acquired at slack water, to reduce any positioning error. At the same location of the sediment samples, a Qumox SJ4000 camera installed on an aluminium frame was dropped on the seafloor to shoot underwater images of each sediment sample site. The fine fraction (<1 mm) was analysed by laser diffraction (Mastersizer 3000, Malvern). The measurement range of the analyser is 0.03–1000 μm. The coarse fraction (>1 mm), mainly composed of shell detritus, was analysed with a mechanical sieve. The samples were classified according to the Folk and Ward method ([66]) using Gradistat statistical package ([67]) and EntropyMax ([68]) software. The photoshoots were all visualised with the VLC software to extract a representative image of the bottom.

### Geomorphometric analysis

The morphological features of the Lido inlet were identified visually within the ArcGIS platform following the classification of shallow coast landforms of [69]. For all the three datasets we generated contour lines with a spacing of 0.5 m. Bathymetric (with a 50% transparency), hill shade and contour layers were overlapped to help the interpretation. Every feature was classified and inserted in a Geodatabase with its dimensions for further analysis. The classification separated erosive and depositional bedforms and, in particular, identified scour holes, dunes and anthropogenic features on the sea-floor. Scour holes can be defined as localised erosional features generated on a sediment surface by a turbulent current ([69]; [70]). Dunes are defined as flow-transverse repetitive bedforms that develop when a sediment bed is subjected to a current ([71]). Anthropogenic features include MoSE main structures, rip-rap areas and dredging marks.

A visual classification is subjective and non-repeatable. Therefore, we implemented semi-automatic and repeatable protocols to identify the morphological features for the three datasets. In this way, we could compare the identified morphologies and assess their changes over time.

#### Scour holes and big dune

*Bathymetric Position Index* (BPI) from the ArcGis toolbox *Benthic Terrain Modeler* (BTM) ([72]) is a second order derivative of the bathymetry ([73]). The BPI algorithm compares each cell’s elevation to the mean elevation of the surrounding cells within a user defined annulus. In the case of crests, a cell will be higher than the surrounding cells in the annulus, giving positive BPI values. Similarly, for depressions the BPI values will be negative ([74]). The BPI tool was used to automatically identify scour holes and a large dune located in the study area. To run the BPI function, the three bathymetric rasters were resampled at 2 m resolution using the *Resample* tool. This operation reduced details of rasters but did not affect the broad-scale morphological identification. The BPI inner and outer radius of the annulus were selected for every scour hole for the dataset 3 by comparing the BPI results with the results of the visual classification (Table 3). In this way the BPI algorithm identified concave and convex areas, respectively.

**Table 3:** BPI identification protocol with the inner and outer radius for each scour and the resulting classes that identify them: Class A = (broad BPI ≤ -3); class B = (-3 < broad BPI ≤ -1); class C = (-1 < broad BPI ≤ 0); class D = (0 < broad BPI ≤ 2) and class E = (2 < broad BPI).

Morphology	BPI values (Inner radius; outer radius)	BPI class
Scour hole S1	15; 250	A
Scour hole S2	15; 500	B
Scour hole S3	15; 150	A
Scour hole S4	15; 250	A and B
Scour hole S5	15; 500	C
Scour hole S6	15; 250	B
Large dune	15; 150	D (2011, 2013); E (2016)

**Fig 2:**
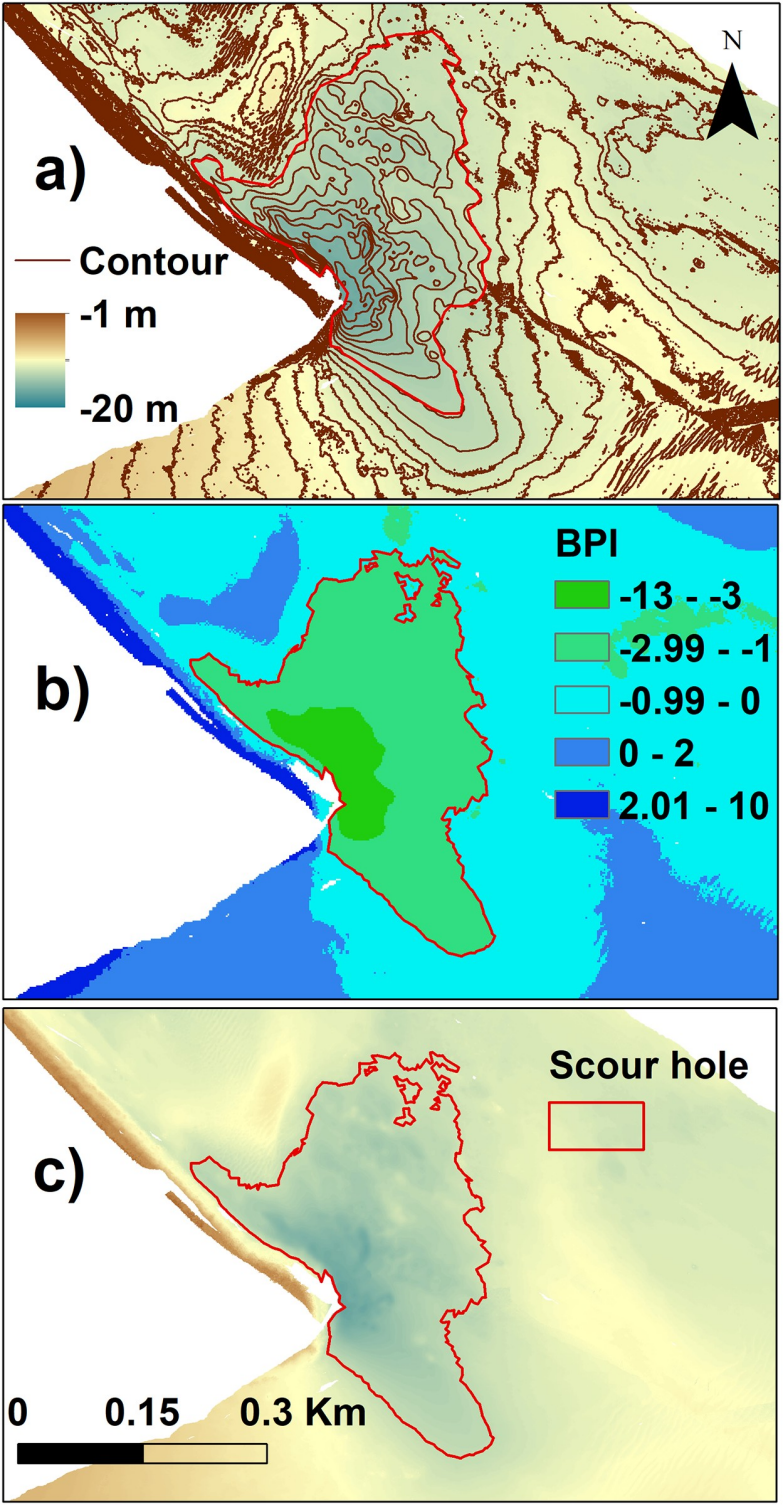
Scour hole identification. a) Bathymetry and contours of the scour hole S4 and the scour hole polygon visually identified; b) result of the broad scale BPI obtained with the BTM; and c) polygon identifying the scour hole S4.

After comparing the BPI values with the results of the visual classification, the broad BPI values were divided into 5 classes (Fig 2 and Table 3): Class A (broad BPI ≤ -3) identified the scour holes S1, S3 and S4; class B (-3 < broad BPI ≤ -1) identified the scour holes S2, S4 and S6; class C (-1 < broad BPI ≤ 0) identified the scour hole S5; class D and E (0 < broad BPI ≤ 2 and broad BPI>2) identified the large dune close to the inlet mouth.

Once the broad BPI class was defined, the following steps were repeated for each dataset: first we applied the *Reclassify* tool to the BPI raster, keeping only the class values that correctly identified the selected feature. This raster class was then converted into a shapefile by *Raster to polygon* tool (Fig 2c). *Calculate areas* was used to calculate the areas of the selected features (see [70]). Scour hole S6 was identified with a semi-automatic procedure: the BPI tool was not able to distinguish between the scour hole and adjacent dredged channel, so this separation was made following the -12 m contour.

#### Dune fields

After several tests with different choices of parameters and classes, by comparing with the visual interpretation, we found that the tool that was better suited for the purpose of automatic identification of dune fields was the *Vector Ruggedness Measure* (VRM or *Terrain Ruggedness*), that can be found inside the *Benthic Terrain Modeler* (BTM) toolbox of ArcGIS ([72]). The VRM calculates the dispersion of vectors perpendicular to each grid cell of the surface and therefore calculates the ruggedness of the sea-floor ([75]). VRM shows low values both in steep and flat areas, but when the floor is both steep and rugged, its values increase. For the seafloor, VRM measures the complexity of the terrain and, in this way, it highlights the presence of an irregular profile ([75]).

**Fig 3:**
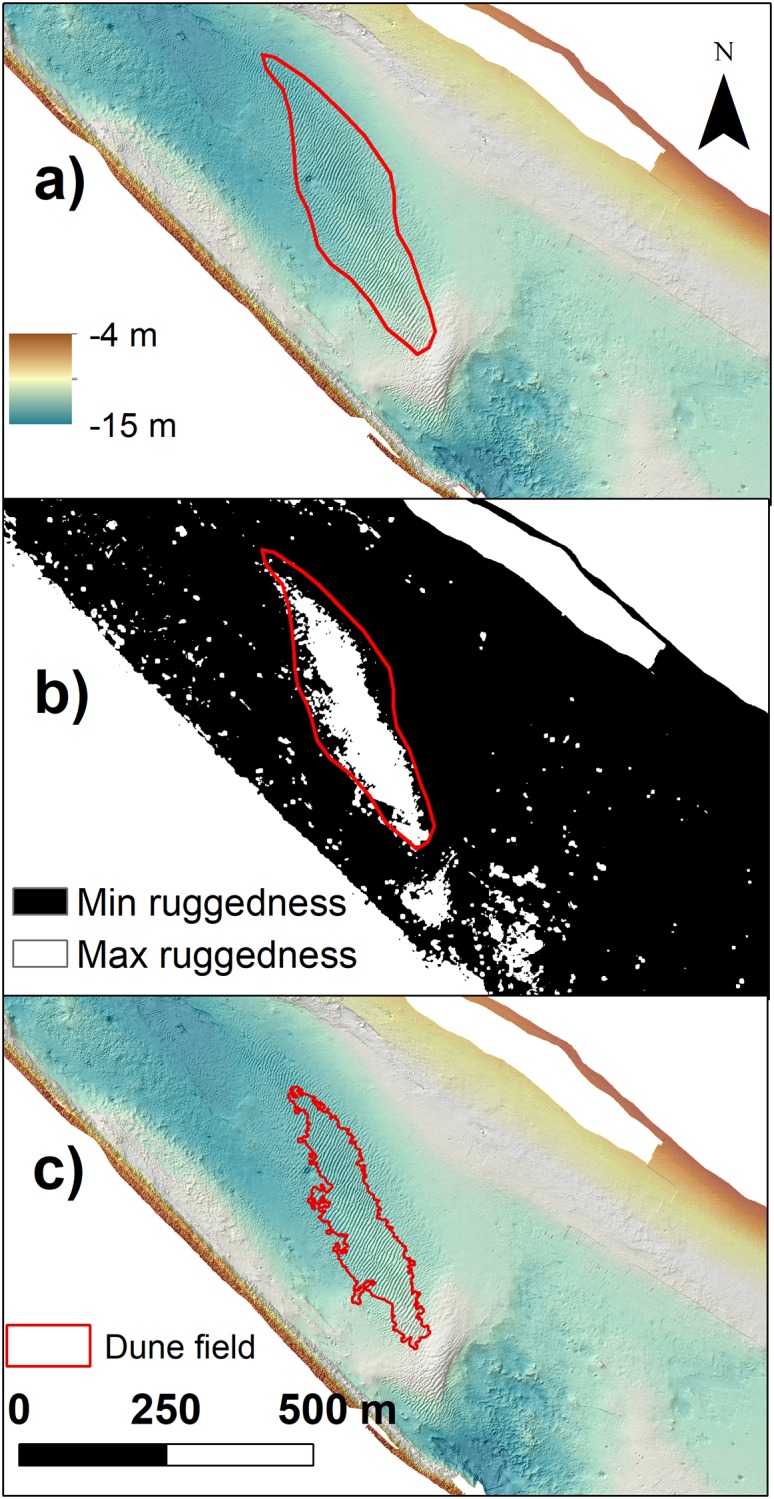
Dune fields identification. a) Bathymetry of the dune field number 1 (Fig 4d) visually identified (red polygon); b) ruggedness map obtained with the BTM; and c) polygon of the dune field extracted from the ruggedness map.

With a bathymetric raster resolution of 0.5 m, VRM was computed by means of a 11 x 11-pixel window (Fig 3). The continuous values from the resulting raster were divided into 2 classes representing minimum (< 0.005) and maximum (>0.005) ruggedness. In this way, we automatically extracted the dune field that was also visually identified (red polygon in Fig 3b).

VRM raster was then reclassified maintaining only the class that represented the dune field. Then the one-class raster was converted into a shapefile with the tool *Raster to polygon*. From this shapefile, it was possible to measure the dune field area with the tool *Calculate areas*.

Another two layers were created to help in the bedform interpretation: *Slope* and *Aspect*, both deriving from the bathymetric layer (with a 0.5 m resolution).

### Volume calculations of bathymetric variations

We calculated the volume differences by integrating the difference of the DTMs over a certain area S. We computed the net sediment volume displaced between any pair of surveys:

$$\begin{array}{c} V\left(S\right)=\sum_{i\in S}\Delta Z(i)A=\sum_{i\in S}[\;Z_{2}\left(i\right)-Z_{1}\left(i\right)]\;A, \end{array}$$ (1)

where V is the volume, S is the area that we analysed, i indicates a grid cell, Δ*Z*(*i*) is the difference of depth value for the grid cell i (i.e. the difference between the depth from survey *Z*_1_(*i*) and the survey *Z*_2_(*i*), in m) and A is the surface area of one grid cell (in *m*^2^), i.e. 0.25 *m*^2^.

The 2016 bathymetric raster lacked data for the scour hole S4 and for other small areas in the whole bathymetry. To overcome this problem, data were interpolated with the tool Focal statistics and so data gaps were substituted using interpolated data.

### Error computation

As pointed out by [76], it is crucial to estimate the uncertainty connected with every areal and volumetric measurement. For this estimate, we selected a common reference area for all datasets. The software CARIS calculated the Total Propagated Uncertainty (TPU) for the reference area, both vertical and horizontal for every dataset, with a confidence level of 95%. The error *σ*_*z*12_ related to the difference of two DEMs (e.g. 1 and 2) was computed as the quadratic propagated error of each DEM $\sigma_{z12}=\surd \left(\sigma_{z1}^{2}+\sigma_{z2}^{2}\right)$, where *σ*_*z*1_ and *σ*_*z*2_ are the vertical TPU values of each DEM used to calculate the difference. To calculate the error in the volume measurements, we applied the formula:

$$\begin{array}{c} \sigma_{V}=\sigma_{z12}\cdot A\cdot N \end{array}$$ (2)

where N is the total number of pixels contained in the morphological feature under consideration.

For the measurements of areas, the formula applied for the error was:

$$\begin{array}{c} \sigma_{area}=\sigma_{h}\cdot A\cdot P \end{array}$$ (3)

where P is the perimeter of the morphological feature under investigation. The vertical and horizontal TPUs for each dataset are collected in Table 4.

**Table 4:** Vertical and horizontal TPUs for each dataset.

Dataset	Vertical TPU *σ*_*z*_ (m)	Horizontal TPU *σ*_*h*_ (m)
**2011**	0.156	0.191
**2013**	0.0987	0.381
**2016 I.I.M**.	0.146	0.191
**2016 ISMAR**	0.119	0.254

### Backscatter analysis

Various methods have been proposed to classify backscatter intensity maps (e.g. [77]; [78]; [47]) in order to identify sub-regions with similar surficial seafloor composition. In this study, given the effectiveness proved by this method in other areas of the Venice lagoon ([40]; [79]), we decided to follow an unsupervised methodology ([77]) and the implementation of the Jenks’ Optimization clustering technique, a tool that can be found in the ArcGis software. Given a user-defined number of classes, the Jenks’ algorithm provides the classification of the backscatter intensity map reducing the variance within classes and maximizing the variance between classes ([80]). We first applied this classification for the 2016 dataset, the only year in which sediment samples were collected.

## Results

**Fig 4:**
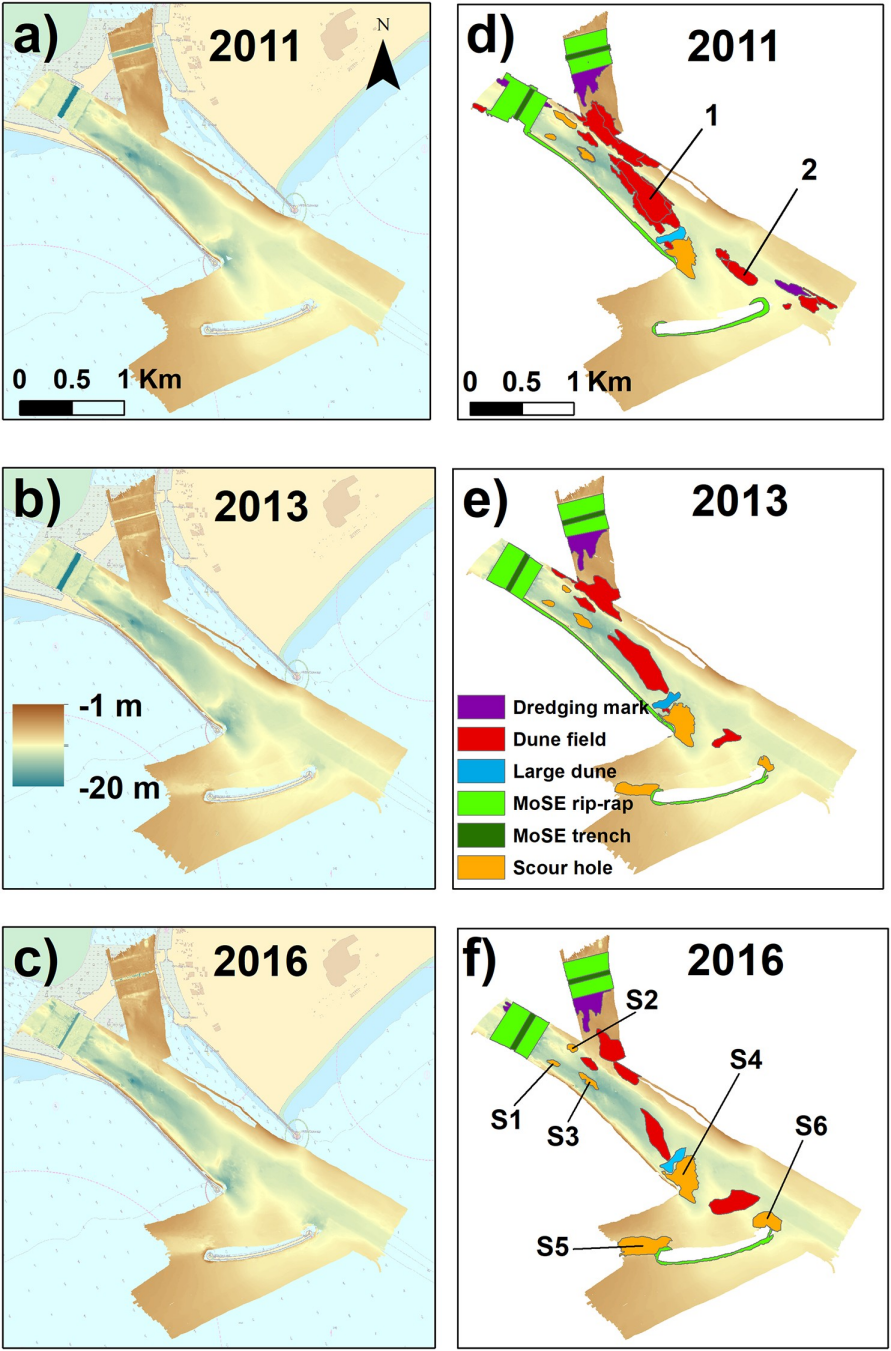
Datasets and morphologies. Left: Bathymetries (with 0.5 m DTM resolution) of Lido inlet collected in a) 2011 (Dataset 1); b) 2013 (Dataset 2) and 2016 (Dataset 3), respectively. Right: Morphological features visually identified for d) dataset 1; e) dataset 2 and f) dataset 3: in green the MoSE structures; in purple the dredging marks; in red the dune fields and in orange the scour holes. Number 1 indicates the dune field pictured in Fig 3 and the number 2 the dune field outside the inlet mouth, whose extension changed its main orientation by about 100°. In figure f) is shown the position of the scour holes. To see the details of the morphologies shown in this image see S2–S4 Figs in the Supporting Information. Reprinted from Nautical Chart 226 under a CC BY license, with permission from Italian Hydrographic Institute, original copyright 2016.

We visually identified the different morphological features for each dataset (Fig 4 and S1–S4 Figs). The left side of Fig 4 shows the DEMs of the study area in 2011, 2013, 2016 whereas the right side highlights the classified morphologies for the different years. The deepest area represents the trench where the MoSE mobile barriers will be positioned (delimited in dark green in Fig 4 right). In 2016, the trench depth decreased both in the south and north branches of the inlet because the mobile barriers were partly set in the position. The trench is surrounded by a rip-rap positioned on the seafloor (light green in Fig 4 right). The presence of a dredged channel is also evident in all three pictures on the south-western side of the inlet, crossing it entirely from the lagoon to the sea. In the north-eastern side of the inlet, instead, the water is shallower (Fig 4 left).

In the classification, we mapped some dredging marks (purple), mainly set close to the MoSE structures (Fig 4 right). The number of scour holes (orange) increased over time: from the 4 depressions visually identified in 2011 (Fig 4d), the number increased to 6 in 2013 and 2016 (Fig 4e and 4f, respectively).

The number of dune fields (red) observable inside the inlet decreased throughout the years (Fig 4 right and S1 Fig); in 2011, 20 dune fields were identified (Fig 4d), in 2013 they fell to 6 and in 2016 only 5 were left (Fig 4e and 4f, respectively). The average properties of each dune field were measured manually and are collected in S1 Table in the Supporting Information.

The large dune at the seaward end of the inlet was recorded in all the three surveys (light blue in Fig 4 right). Given that scour holes and dune fields appear as the most dynamic features, we applied the geomorphometric analysis described before to map them semi-automatically: the scour holes and the large dune using the broad BPI; the dune field depicted in Fig 4d(1) using the VRM classification. In fact, we found that the VRM terrain attribute can identify univocally only the dune fields with wavelength (λ) between 5 and 6 m. For this reason, to compare the dune fields recognised with this method, we needed this wavelength to be maintained throughout the whole period (2011-2016). The only dune field that satisfied this was the one represented in Fig 4(1).

**Fig 5:**
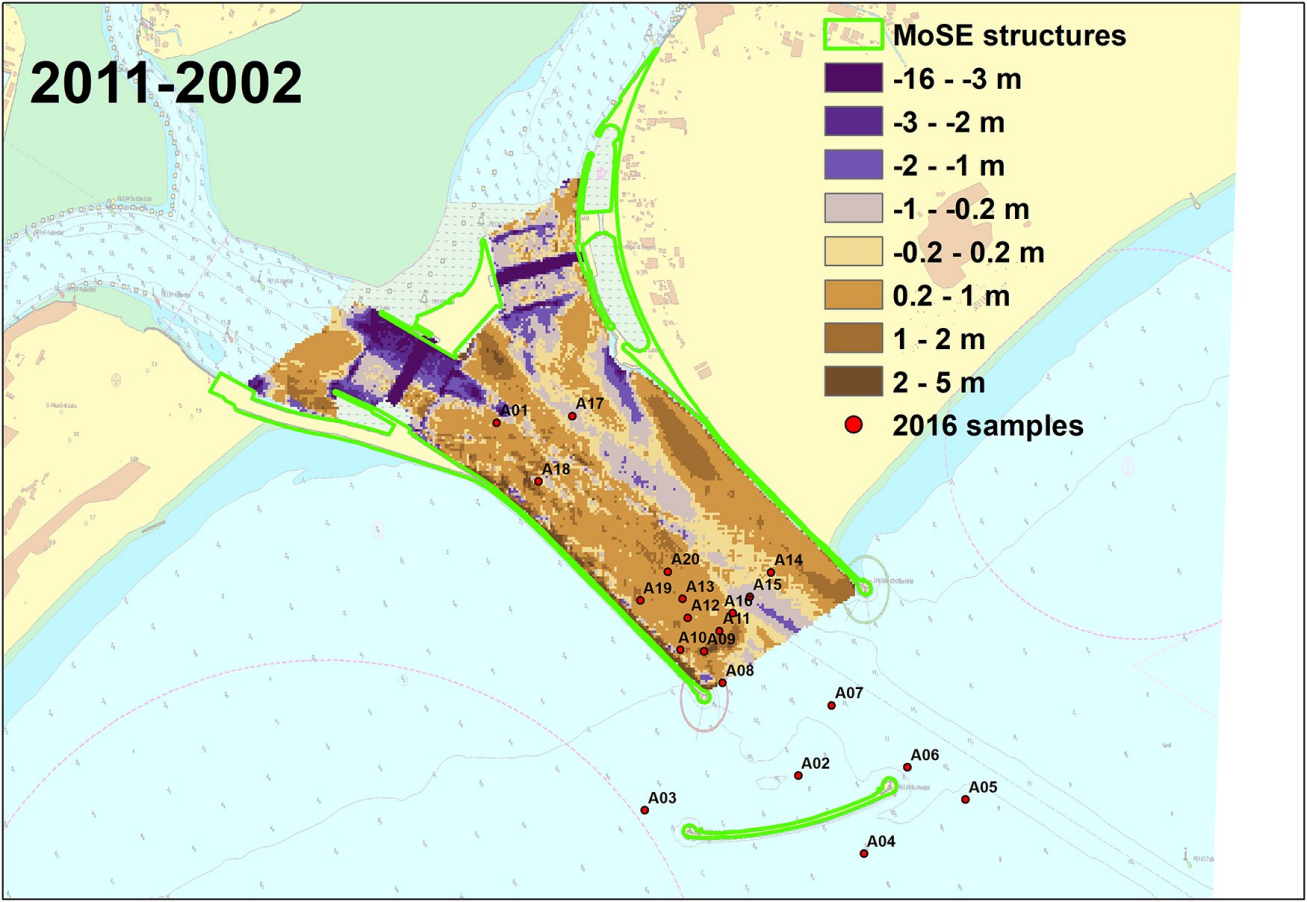
Changes due to the MoSE construction. Qualitative bathymetric difference between a 2002 bathymetry (before the MoSE construction) and the 2011 survey (after the MoSE construction). The green polygons show the new structures built for the MoSE since 2003. Reprinted from Nautical Chart 226 under a CC BY license, with permission from Italian Hydrographic Institute, original copyright 2016.

After the comparison of the inlet morphological features, in order to understand the evolution of the whole tidal inlet over time, we first compared the satellite images of the inlet of 2004 and 2017 (Fig 1 right) to estimate the surface occupied by the new structures (in green in Fig 5). The comparison shows that the surface of the inlet inside the channel was reduced by about 437200 *m*^2^, whereas the breakwater occupies 34013 *m*^2^. Then, we qualitatively compared the latest complete low-resolution bathymetry of the Venice Lagoon collected in 2002 ([81]) before the MoSE works even started (before MoSE) and our MBES bathymetric map of 2011 when the construction of the MoSE structures was all finished (after MoSE). The residual bathymetry is shown in Fig 5. The North-Eastern channel, which is shallower than the South-Western challange (average depth of 6 m versus 12 m), was eroded more, as testified by the prevalent blue colour in Fig 5. The trenches corresponding to the lodgement of the mobile barriers were deepened more than 10 m. The area that became deeper (more than 3 m) is close to the rip-rap around the lodgement of the mobile barriers.

Overall, the inlet seems to be in deposition at the northern and southern flanks of the navigation channel, that became deeper.

**Fig 6:**
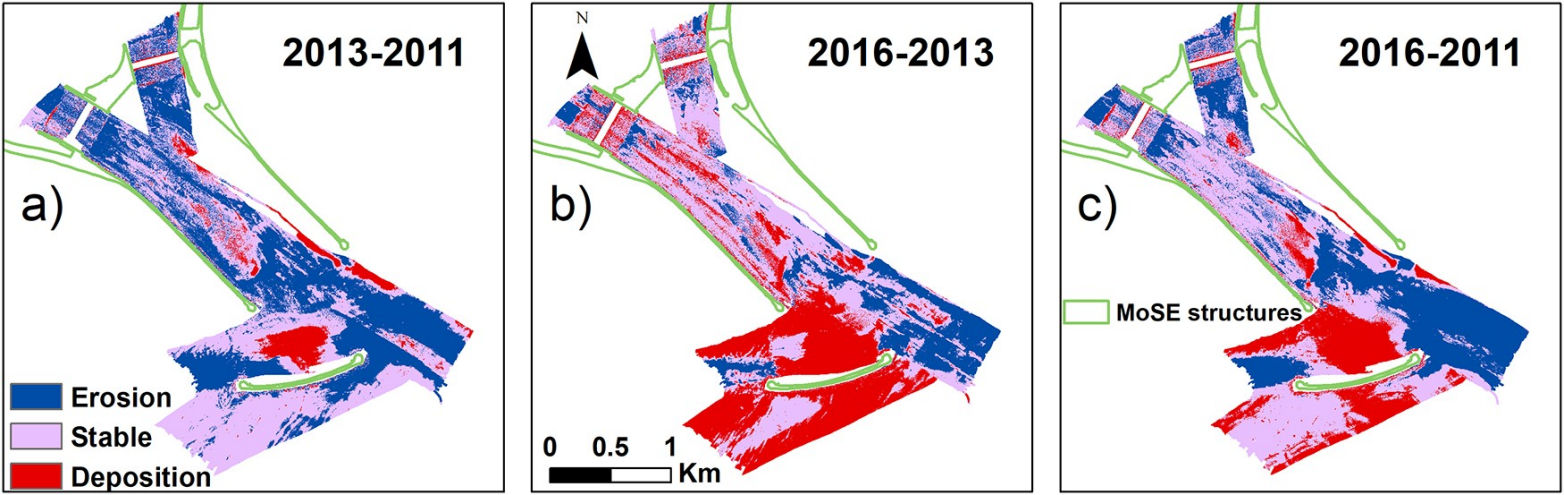
Bathymetric differences between any pair of surveys. a) 2013-2011; b) 2016-2013 and c) 2016-2011. Blue colour: areas affected by an erosional process; red colour: areas where the depositional process was predominant; grey colour: areas where no detectable change has occurred.

To quantitatively estimate the more recent changes of the inlet, we compared the DEMs of each MBES survey from 2011 to 2016, assuming that areas with a bathymetric difference outside the error interval can be considered in deposition or erosion, depending on the sign of the difference. The areas with difference values inside the error interval can be considered stable. The bathymetric and volume differences between any pair of surveys are shown in Fig 6 and in Table 5, respectively: in blue are the areas under erosion, in red the areas in deposition, while in grey are the stable areas. A similar figure with the polygons of the morphologies and the raster of differences can be found in Supporting Information (S5 Fig).

**Table 5:** Volume differences of the areas under erosion and deposition in the whole study area, extracted by comparison of the datasets 1, 2 and 3 and relative errors.

	Volume difference 2013-2011 (10^3^*m*^3^)	Volume difference 2016-2013 (10^3^*m*^3^)	Volume difference 2016-2011 (10^3^*m*^3^)
**Erosion**	-864.7 ± 38.4%	-341.9 ± 30.0%	-1016.8 ± 22.5%
**Deposition**	138.1 ± 39.0%	549.3 ± 33.1%	404.6 ± 31.3%
**Net**	-726.6 ± 46.3%	207.3 ± 100.7%	-612.2 ± 42.7%

During the first two years (2011-2013—Fig 6a), we observe a prevalent erosion with an overall net sediment loss of 726.6 · 10^3^ ± 46.3% *m*^3^ (Table 5). The deposition (138.1 · 10^3^ ± 39.0% *m*^3^) predominantly occurred at the lagoon-side of the newly-built offshore breakwater. From 2013 to 2016 (Fig 6b), instead, the deposition process was dominant (549.3 · 10^3^ ± 33.1% *m*^3^) with a net sediment gain of 207.3 · 10^3^ ± 100.7% *m*^3^ (Table 5). Over about five years, from June 2011 to September 2016, there was a net sediment erosion of 612.2 ± 42.7% *m*^3^ (Fig 6c and Table 5). Also in this case, like in the period 2002-2011, the North-East channel seems to experience a more severe erosion than the South-West channel.

### Scour holes

Scour holes S1, S2 and S3 are located inside the inlet (Fig 4f); scour hole S4 is located at the seaward end of the southern jetty; scour holes S5 and S6 are sited at the south and north ends of the breakwater located outside the inlet, respectively.

**Fig 7:**
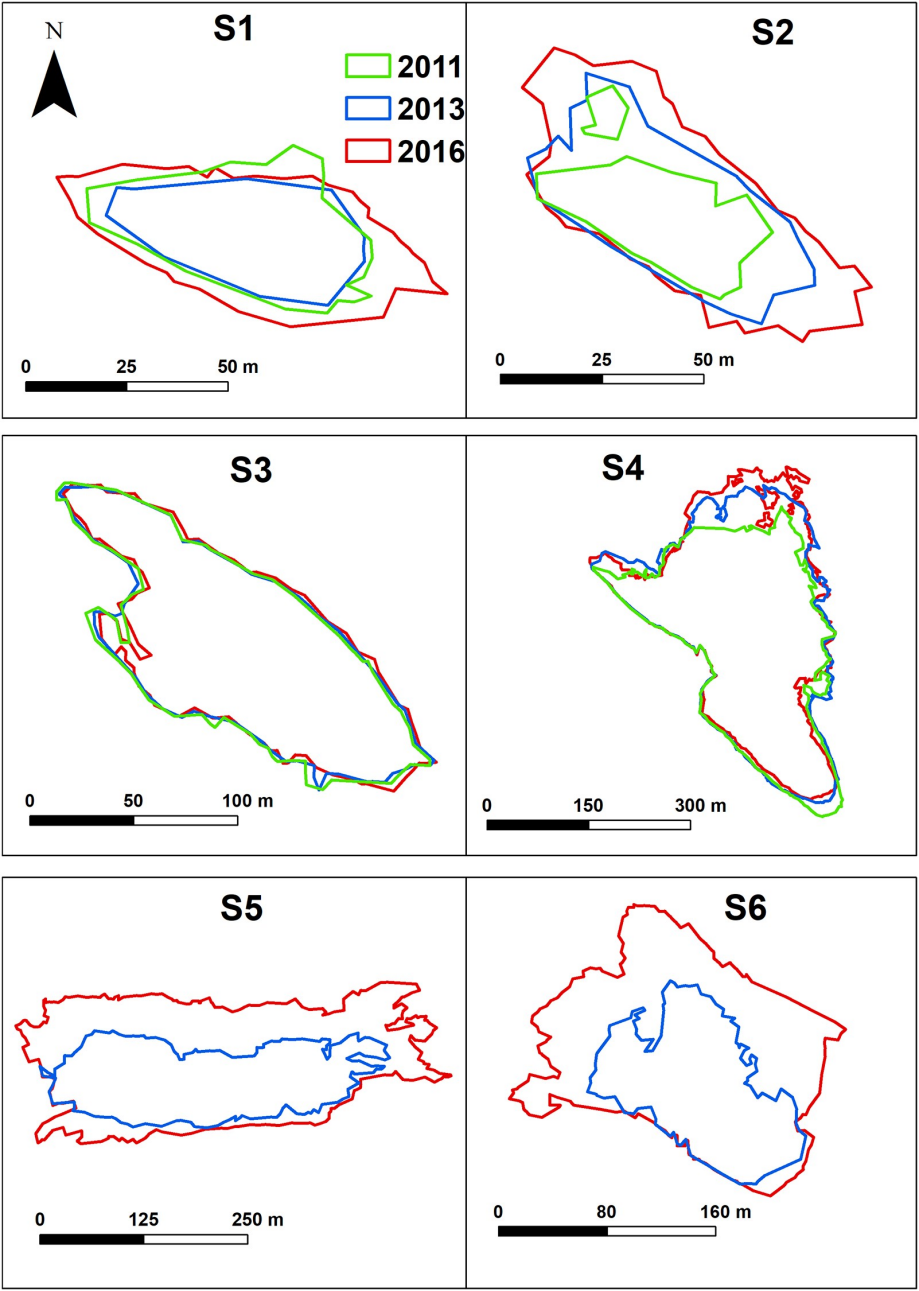
Scour holes evolution. Evolution of the scour holes extent during the 5 years: green, blue and red polygons indicate the extent of the scour holes in the years 2011, 2013 and 2016, respectively.

Fig 7 shows the green, blue and red polygons that identify the scour holes in 2011, 2013 and 2016, respectively. Starting from the DEMs we computed the areas of the scour holes and the volume differences over the years, with relative errors (Table 6).

**Table 6:** Areas of the scour holes in the different years and relative errors; volume difference extracted comparing the datasets 1, 2 and 3 and relative errors; maximum depth of the scour holes in 2016.

Morphology	area (10^3^*m*^2^)			Volume difference (10^3^*m*^3^)			Maximum relative depth (2016) (m)
	2011	2013	2016	2013-2011	2016-2013	2016-2011	
**Scour hole S1**	1.7 ± 2.1%	1.4 ± 4.4%	2.3 ± 1.9%	-0.2 ± 111.6%	0.3 ± 147.4%	-0.1 ± 411.8%	1.4
**Scour hole S2**	1.2 ± 3.2%	2.0 ± 3.9%	2.9 ± 1.7%	-0.6 ± 63.2%	-0.3 ± 165.1%	-1.2 ± 49.7%	1.1
**Scour hole S3**	11.3 ± 1.0%	11.1 ± 1.9%	11.1 ± 1.1%	-1.4 ± 147.0%	0.3 ± 685.4%	-1.1 ± 209.8%	4.9
**Scour hole S4**	74.6 ± 0.4%	85.6 ± 0.8%	83.4 ± 0.5%	-30.4 ± 50.6%	11.0 ± 163.4%	-22.8 ± 76.4%	6.1
**Scour hole S5**	-	32.3 ± 1.5%	66.3 ± 0.5%	-58.1 ± 10.0%	-23.8 ± 49.9%	-114.3 ± 12.1%	4.1
**Scour hole S6**	-	12.3 ± 2.1%	27.6 ± 0.6%	-21.7 ± 10.1%	-18.9 ± 26.2%	-58.2 ± 9.9%	2.9

The only scour hole whose shape remained constant over the studied interval was S3 (Fig 7). All the other depressions showed a change in size and shape. In detail, scour hole S5 and S6 did not exist when the first dataset was collected (2011), they appeared in 2013, changing consistently their shape and extent over time. Their areas doubled from 2013 to 2016 (S5: from 32.3 · 10^3^ ± 1.5% *m*^2^ to 66.3 · 10^3^ ± 0.5% *m*^2^ and S6: from 12.3 · 10^3^ ± 2.1% *m*^2^ to 27.6 · 10^3^ ± 0.6% *m*^2^) (Table 6). We computed that the newly formed scour holes (S5 and S6) increase their area at a speed of 14215 *m*^2^/year and 5923 *m*^2^/year respectively.

The differences in volume during the five years show a pronounced erosion of the scour holes S5 and S6 (Table 6), recording a sediment loss of 114.3 · 10^3^ ± 12.1% *m*^3^ from 2011 to 2016 in the scour hole S5 and of 58.2 · 10^3^ ± 9.9% *m*^3^ in the scour hole S6. The other scour holes (S1, S2, S3 and S4) show irrelevant changes in their volumes, below the estimated error (Table 6).

### Dune fields and large dune

**Fig 8:**
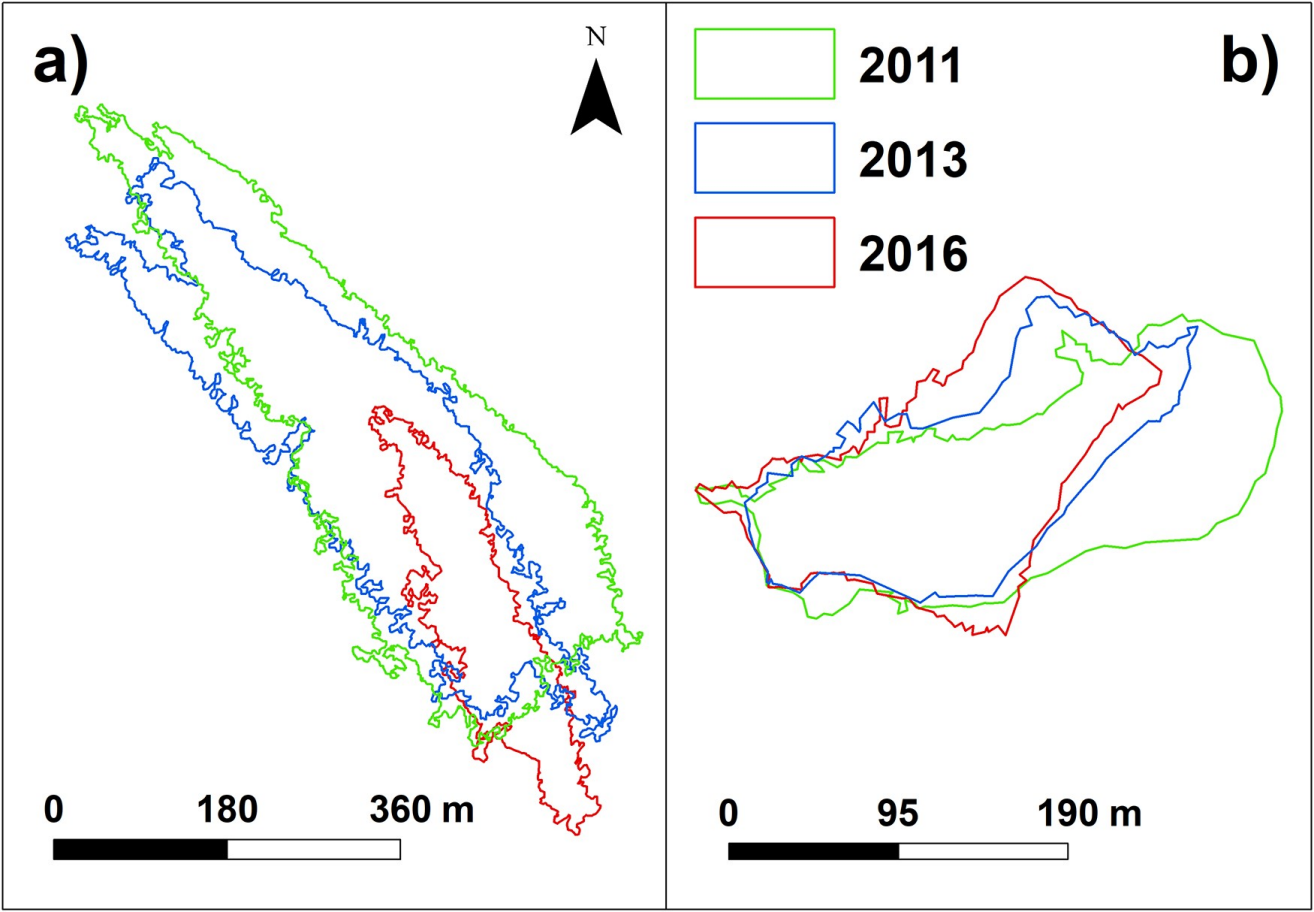
Dune fields variation. a) Change over 5-year time of the dune field extent and b) of the large dune, whose positions are highlighted in Fig 4 right: green, blue and red polygons indicate the extent of the morphologies in the years 2011, 2013 and 2016, respectively.

The area of dune fields shows a drastic decrease. In 2016, it is less than half of the surface of 2011: from 437.9 · 10^3^ ± 0.1% *m*^2^ in 2011 to 256.0 · 10^3^ ± 0.2% *m*^2^ in 2013 and, finally, to 212.5 · 10^3^ ± 0.1% *m*^2^ in 2016 (Table 7). The average reduction rate is of 48298 *m*^2^/year. In particular, the dune field identified with the VRM method (Fig 8a) reduces its area from 122.7 · 10^3^ ± 0.2% *m*^2^ in 2011 to 92.4 · 10^3^ ± 0.5% *m*^2^ in 2013 and to 32.5 · 10^3^ ± 0.3% *m*^2^ in 2016 (Table 7). Its original area shrunk by about 75% at a speed of 19337 *m*^2^/year.

**Table 7:** Areas of the dune fields and large dune in the different years and relative errors; volume difference extracted comparing the datasets 1, 2 and 3 and relative errors.

Morphology	area (10^3^*m*^2^)			Volume difference (10^3^*m*^3^)		
	2011	2013	2016	2013-2011	2016-2013	2016-2011
**Large dune**	27.5 ± 0.7%	20.7 ± 1.5%	22.7 ± 0.7%	-2.4 ± 156.6%	5.5 ± 74.8%	3.3 ± 144.0%
**Dune fields**	437.9 ± 0.1%	256.0 ± 0.2%	212.5 ± 0.1%	-	-	-
**VRM dune field**	122.7 ± 0.2%	92.4 ± 0.5%	32.5 ± 0.3%	-	-	-

Fig 8b shows the variation in extent of the large dune, identified with the BPI procedure. The large dune stepped backwards and rotated, changing its main axis from West-East to South West—North East from 2011 to 2016. In Table 7 the area and volume differences between surveys are reported. In the period 2011-2013, the large dune area decreases from 27.5 · 10^3^ ± 0.7% *m*^2^ to 20.7 · 10^3^ ± 1.5% *m*^2^. In the following three years, it increases from 20.7 · 10^3^ ± 1.5% *m*^2^ to 22.7 · 10^3^ ± 0.7% *m*^2^.

The volume differences show no relevant change and remains below the estimated error during the five years (Table 7).

### Seafloor sediment distribution

#### Sediment grain size

The 2006 samples were collected close in time with the MBES survey, to minimize the possibility of a change in the seafloor composition. A transect of 5 samples was collected at the inlet mouth, one sample on the bottom of the three main scour holes, one at the lagoon-side of the breakwater and the remaining 11 samples on the dune fields (Fig 5). The Gradistat analysis classified the samples according to the Folk and Ward diagram ([66]) as: Sand, Slightly Gravelly Sand, Gravelly Muddy Sand, Gravelly Sand, Muddy Sandy, Sandy Gravel, Gravel and only one as Sandy Mud (S1 Table). To better enhance the grain size variation between samples, a cluster analysis (EntropyMax) was applied. The 20 samples were grouped in 8 groups.

#### Backscatter classification

**Fig 9:**
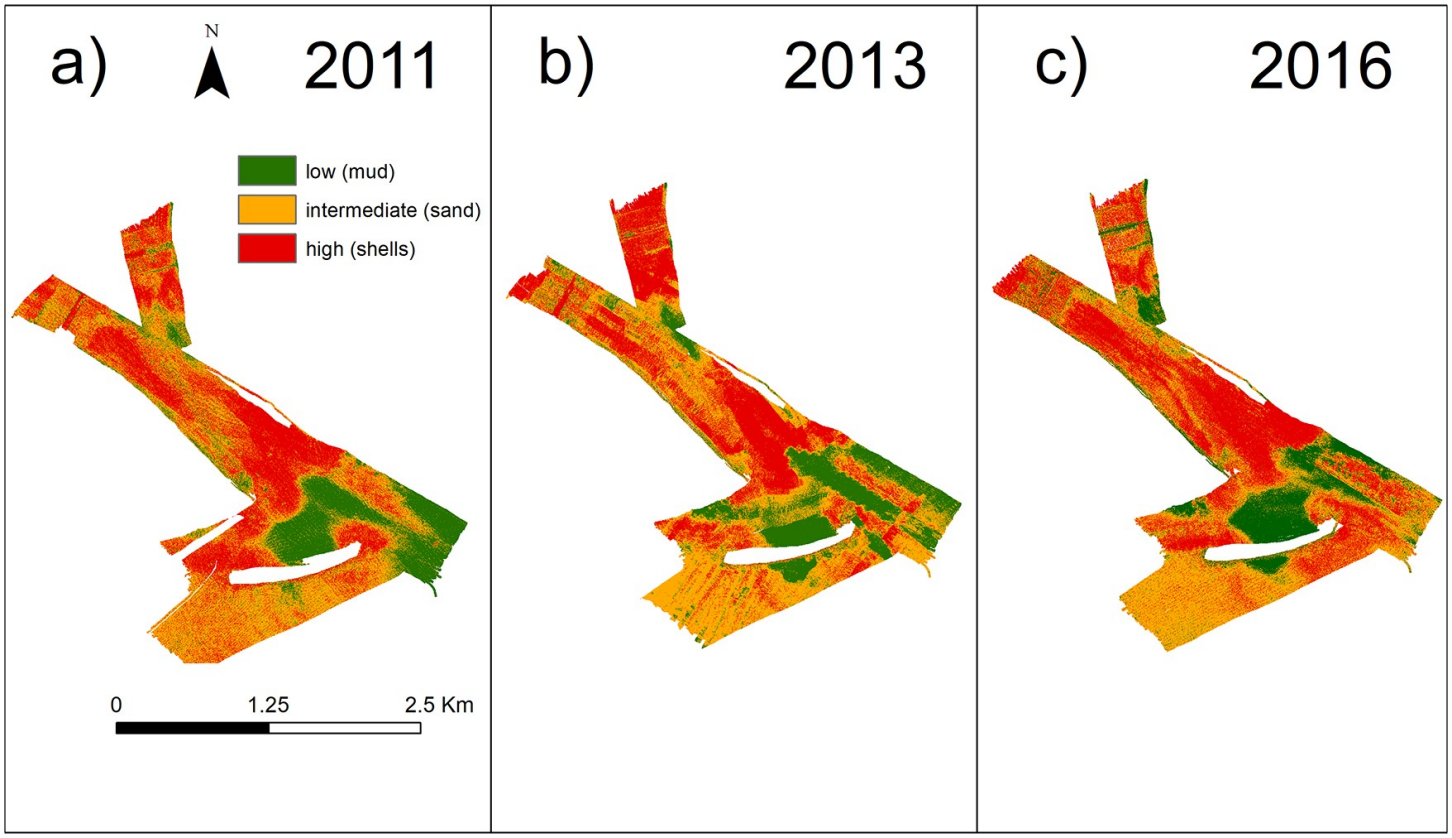
Backscatter comparison. Comparison of the classified backscatter in a) 2011, b) 2013 and c) 2016.

It was already demonstrated that backscatter intensity is related to sediment particles dimension: coarse sediment is characterised by higher backscatter intensity values, while fine sediment correspond to lower backscatter intensity values ([82]; [83]; [84]; [26]). By comparing the backscatter distribution and the grain size analysis, we found a good agreement between samples and backscatter classification by rearranging the 8 groups of samples in three classes, corresponding to the three backscatter classess obtained thanks to the Jenks’ optimization clustering technique. The three backscatter classes (Fig 9) identify low, intermediate and high backscatter intensity (< -30.24 dB, between -30.24 dB and -24.43 dB, > -24.43 dB respectively).

The three classes obtained combining the backscatter and the grain size results (S6 Fig), were named after the prevalent type of sediment found on the seafloor, as follows ([85]):

- Class I (muddy sediment): this class is represented by the only muddy sample collected at the lagoon side of the breakwater (main mode ∼ 22 *μ*m). The analysis of the images shows the complete absence of shells on the seafloor.
- Class II (mainly sandy sediment): this class is characterised predominantly by well sorted sand (72.5%—100% of sand, main mode ∼ 250 *μ*m).
- Class III (gravelly sediment): this class corresponds to an high content of shells (> 33%). In this case the grain size curve has two modes at ∼ 250 μm and ∼ 8000 *μ*m. The analysis of the images shows the seafloor completely covered by shells and shell detritus.

We found that this unsupervised Jenks’ classification shows an overall accuracy of 75% ([85]) obtained deriving the confusion matrix (S2 Table) and counting the percentage of correctly allocated cases ([86]). The same Jenks’ classification was applied by analogy to the 2011 and 2013 datasets. This procedure allowed a qualitative comparison of the classified backscatter images obtained for each survey. Overall we observed the following main changes in the seafloor sediment distribution: at the lagoon-side of the breakwater, from 2011 to 2016, a muddy area has become bigger, while inside the inlet we observed a change in sediment composition, from mainly sandy to mainly gravelly, as shown in Fig 9c).

## Discussion

Fontolan et al. [62] defined the Lido inlet as stable, whereas Tambroni and Seminara [87] stated that the inlet was prone to deposition. Helsby [55] demonstrated that from 1930 to 2000 the northern lagoon channels were experiencing higher rates of deposition than of erosion. Lido inlet, in particular, was considered depositional. Defendi et al. [88] measured the total transport in the Lido inlet based on calibrated ADCP measurements in 2005-2006 and by coupling the transport model SEDTRANS96 ([89], [90]) to the SHYFEM hydrodynamic model ([91]; [92]), showing a sediment loss of 381 ⋅ 10^3^ *m*^3^/year for the lagoon and of 256 ⋅ 10^3^ *m*^3^/year for the Lido inlet, with a bed-load transport of 29 ⋅ 10^3^ *m*^3^/year. These studies referred to the inlet before the most recent modifications.

In Fig 5, we show that in the period between 2002 (before MoSE) and 2011 (after MoSE) in the Lido inlet the deposition was still prevalent, except for the area close to the new island and the navigation channel, that is periodically dredged. The sediment transport observed in [88] could be related mainly to the erosion of these areas.

Using high-resolution MBES, we then quantitatively estimated changes in the time span of almost five years (2011-2016) immediately after the construction of the island in the inner part of the inlet and contemporary to the building of the offshore breakwater (2010-2012).

By comparing the bathymetric survey of 2011 and 2016 (Fig 6c), we found a dominant erosive trend with a net volume loss of 612.2 · 10^3^ ± 42.7%. The prevalent erosion found in the entire period 2011-2016 is probably due to the engineering works carried out in Lido inlet since 2004 for the MoSE project: the change in the inlet configuration and the construction of the island likely increased the current speed in the inlet channel, as shown in the modelling simulations of [93], with a consequent increase in the bottom shear stress and sediment resuspension, reallocation and possible transport outside the inlet. We compared the sections of the tidal inlet in 2016 and in 2002 with and without the presence of the island. The area resulting from the sum of the two sections (North and South channels) extracted from the 2016 bathymetry (after the construction of the island) is about 67% of the section measured in 2002. The section of 2002 was about 10500 *m*^2^ and the channel was anyway separated in two areas (North and South) by a shallow area in the middle that now is occupied by the island. The Northern channel had a section of about 3811*m*^2^ and the Southern channel had an area of about 6746 *m*^2^. In 2016 the section of the northern channel was about 2442 *m*^2^ and the Southern was 4615 *m*^2^ for a total of 7057 *m*^2^. The sections have the same proportions in 2002 and 2016, with 1/3 of the water passing through the northern channel and 2/3 of the water passing through the southern channel. A narrower cross section induces higher current velocity leading to more erosion in the inlet. This could be a simple explanation for the erosive trend in some areas of the inlet channel. Moreover, analysing the data of wave height and water level (see S7 Fig in Supporting Information) in the period 2011-2016, we observe that there were two storm surges of 143 cm on November 1, 2012 and 143 cm on February 12, 2013, one of them just before the survey of 2013. A very high-water level (*acqua alta*) would increase tidal prism, discharge in the inlet, velocities, producing erosion in the tidal channel that is clearly visible in Fig 6a. On the contrary, there were not large acqua alta events between 2013 and 2016. This would explain a return to normal conditions with a slow silting that we observe Fig 6b. This is also confirmed by the comparison of the sediment distribution over time obtained by the backscatter and grain size analysis: in the inlet channel, the mainly sandy sediment is replaced by mainly gravelly sediment (shell and shell detritus).

However, if we just consider the last interval 2013-2016, the volume of deposited sediment is almost four times higher with respect to the previous interval 2011-2013 and the volume of eroded sediment decreases by 2.5 times (Table 5). This shift from erosion to deposition could be related to the construction of the offshore breakwater, since the seasonal changes in erosion and deposition are unlikely to play an important role: in fact, all the surveys were carried out in the same season (summer). This hypothesis seems to be confirmed by the fact that the deposition concentrates close to the hard-coastal structure (Fig 6). Fig 9 shows that in this area mainly mud deposition occurred.

The construction of the offshore breakwater led also to the formation of the two scour holes (S5 and S6) located at its south and north edges respectively (Fig 4f). These scour holes eroded the ebb-tidal delta, as the breakwater was built on top of it. Ebb-tidal deltas are lobes of sediment that accumulates seaward of the inlet and form because of the interaction of tidal and wave-generated currents ([94]; [95]). They are usually found in tidal inlets of most barrier island complexes around the globe ([94]). This feature was completely mapped in the 2011 survey, but not entirely included in the 2013 and 2016 surveys.

Scour holes S5 and S6 increased their maximum depth of more than 2 m over five years. The scouring process is highlighted in Table 6, where the volume differences show a sediment loss both in S5 and S6. The scouring process took place with different velocities, higher for the years 2011-2013, lower for the years 2013-2016: in the two-year-period 2011-2013 the erosion proceeded at 33205 *m*^3^/year and 12405 *m*^3^/year for S5 and S6, respectively. In the first-time interval, the breakwater was still under construction, substantially altering the system equilibrium, with consequently very high erosion rates. These rates reduced in the following three years reaching almost half of the previous values (8158 *m*^3^/year for S5 and 6471 *m*^3^/year for S6). In 2013 the building of the breakwater was completed, and the scour holes seemed to have adjusted to the new configuration with a consequent slowing down of the erosion process. This is in agreement with observations of the scouring process in correspondence of offshore structures, where there is an exponential deepening of the scours after the structure installation ([96]; [97]).

Scour holes occurring around breakwaters have been observed globally, like for example in Japan ([98, 99]), in The Netherlands ([100]) and in the U.S. ([101]). Processes leading to the formation of scour holes around hard coastal structures have been extensively studied mainly on the basis of tank experiments ([20]; [102]; [103]; [104]). Fredsøe and Sumer ([102]) investigated the scouring at the round head of a rubble-mound breakwater by using regular waves. They found that the major mechanism responsible for the scouring is the formation of lee-wake vortices in each half period of the waves. The scouring process is governed by the Keulegan-Carpenter number, KC, which depends on the base width of the breakwater head. Larger values of KC imply the formation of larger scour holes. In our case, we determined KC from Eq 6 of [20]: *KC* = 1 + ((*L*/1.75*B*))^2^, obtaining *KC* = 1.16 with the width of the breakwater *B* = 70 m and the width of protection layer (rip-rap) on the seafloor *L* = 50 m. This value of KC corresponds to a separated flow regime with no horse-shoe-vortex formation in front of the breakwater.

The shape of the scour holes we found is very similar to the scour holes due to non-breaking waves described by [20] at the head of a vertical breakwater. From 2012 to 2016, the monthly averaged wave height and period in the study area ranged from 0.3 to 1.55 m and from 3 to 6.2 s ([105]). The presence of co-directional currents likely contributed to the wave action enhancing the depth of the scour holes, given that large-scale vortices generated at the breakwater tip can increase the transport capacity of the flow.

We estimated that the ratio *d*_*max*_/*h* (relative maximum scour depth/water depth) is equal to 0.5 for the scour hole S5 and 0.2 for the scour hole S6. These values were found for wave-dominated scour holes ([106]). For these reasons, assuming that this ratio will not increase substantially in the near future and being aware that this is a qualitative comparison, it is reasonable to consider that these scour holes are wave-dominated, even though other factors like currents and local bathymetry may play an important role. However, a 3D hydrodynamic modelling analysis would be required to fully understand the role of currents and waves in the scouring process. This process may lead to the gradual dislocation of the rubble mound foundation at the breakwater toe and could progressively endanger the stability of the structures ([107]; [103]).

Several studies applied geomorphometric semi-automated techniques to describe dune fields parameters as wavelength, height, crest orientation ([108]; [109]) or to evaluate their migration ([110]; [111]; [112]). Repeated multibeam bathymetric mapping was used to measure the bed elevation variations caused by dune migration and to estimate the sand transport rates in different parts of the world (e.g. Duffy et al., [110] and [111], in the Bay of Fundy, Canada; Ernsten et al. [113] in the Danish Wadden Sea; Nittrouer et al. [114] in the Mississippi river). More recently, Fraccascia et al. ([45]) studied the morphology and hydrodynamics of the natural tidal inlet of Knudedyb, Danish Wadden Sea, relating the bedforms migration patterns to residual currents. In some cases, geomorphometric analysis of dune fields was crucial to prevent hazards related to human activities (e.g. navigation, construction of pipelines) ([115]; [112]).

In the Lido Inlet, we classified the dune fields by measuring their average properties from the bathymetry (see S3 Table in Supporting Information). From the backscatter classification (Fig 9) we observed that their composition was mainly gravelly in the throughs and sandy on the crests, in agreement with [79]. The direct comparison of the dune properties, however, was not possible since many dune fields disappeared from one survey to the next. In 2011 there were 20 dune fields, whereas in 2016 only 5 were left, with an overall shrinking of the dune field extent of more than half (51.5%) over about five-years (Table 6).

This shrinking of the dune fields occurred at different speeds: between September 2011 and June 2013, the dune fields diminished at a rate of 103916 *m*^2^/year; between June 2013 and May 2016 the rate was of 14927 *m*^2^/year, considerably lower than before. All the surveys were carried out in summer, so it is unlikely that these changes are due to seasonality. They could be related instead to the construction of the island inside the inlet that, as observed before, likely increased the current velocity ([93]). Higher currents and higher bottom shear stresses possibly induced a rapid reduction of the dune fields. This reduction was accompanied by a coarsening of the sediment in the inlet channel. The same happened in the southernmost inlet of the Venice Lagoon ([79]. When the breakwater was built (2013), the overall dune fields surface reduction slowed down. For the dune field shown in Fig 8a, however, the reduction speed increased from 17309 *m*^2^/year in the interval 2011-2013, to 20554 *m*^2^/year in the following three years. At this rate of reduction, this dune field will eventually disappear in less than two years.

The disappearance of the dunes can be explained by the change of shape of the inlet related to the MoSE defence: a) the construction of the island at the land-ward tip of the inlet channel determined a bifurcation of the flux entering the lagoon and a confluence of two channel exiting the lagoon with probably high current velocities that contributed to the erosion of the dune fields close to the mobile barrier structures and in the centre of the inlet; b) the construction of the breakwater determined another flow separation: part of the flow deviates south of the inlet mouth while the other part continues straight.

Consequently, the dunes reduction decelerated, and the orientation of the dune fields changed over time (Figs 4 and 8). In particular, the dune field immediately outside the inlet mouth (indicated by the number 2 in Fig 4d), in 2011 extended parallel to the jetties as was the flux without the breakwater and was about to disappear in 2013. After the construction of the breakwater it was recreated in a south-west direction, with the result that in 2016 a dune field area is still present with an orientation rotated by more than 125° clockwise with respect to the one of 2011.

The general shrinking of the dune fields suggests that the sediment transport regime should have changed radically with the completion of the construction of the artificial island for the MOSE project with an enhanced seaward flux of sand. This is evident by comparing the sediment distribution over time (Fig 9).

This is in agreement with the findings of [116], that analyzed the tidal components in the Northern Adriatic Sea and in the Venice Lagoon during the last 70 years. They observed an increase amplitude of the major tidal components and a shift of the Venice Lagoon tidal asymmetry towards ebb dominance. Particularly, in the last few years, they observed that the recent reduction of the inlets cross-sectional area further enhanced the ebb dominance over the whole lagoon and probably increased the seaward flux of sand, as well. The sand flux through the inlets is dominated by bed-load transport as it was shown by the direct measurements in the Lido Inlet in 2006 (before the construction of the island), by [117]. The disappearing of dune fields may imply the overall reduction of sand transport inside the inlet.

## Conclusion

Tidal inlets are extremely dynamic environments governed by natural processes which control their morphological variations. Often these changes represent a problem to human activities and many actions are taken to limit this natural phenomenon. This is the case of the Lido inlet (Venice, Italy) that went through several human-induced modifications in the last century.

Our investigation suggests that the construction of the mobile barriers (MoSE project) in the last 15 years contributed to the substantial modification of the inlet morphology and sedimentary regime.

Until 2011, different studies and the qualitative comparison between the 2011 and 2002 bathymetries presented in this research showed that the Lido inlet experienced deposition. High-resolution maps of the seafloor (0.5 m) collected in 2011, 2013 and 2016 made possible to compute bathymetric variations and relative errors and to observe underwater morphologies and seafloor sediment distribution in great detail. The comparisons of bathymetries highlighted different sedimentary regimes: mainly erosive from 2011 to 2013 and depositional from 2013 to 2016, with an overall net loss of sediment of 612.2 · 10^3^ ± 42.7% *m*^3^ in five years. Moreover, the overall reduction of depositional areas is accompanied by a general coarsening of the seafloor sediment and by an increase of the number of erosional features.

In detail:

1. Two new scour holes formed at the tips of the recurved breakwater positioned on the seaward side of the inlet. From the comparison with literature on scour holes at breakwaters, we found that these scour holes are more likely wave-dominated than current dominated. During this 5-years research, both these depressions deepened and widened: S5 area went from 32.3 · 10^3^ ± 1.5% *m*^2^ to 66.3 · 10^3^ ± 0.5% *m*^2^; S6 area from 12.3 · 10^3^ ± 2.1% *m*^2^ to 27.6 · 10^3^ ± 0.6% *m*^2^. Scouring took place with two different speeds: faster in the first two year, slower in the following three years. These different velocities are explained by the fact that in the first two-year-period the breakwater was under construction, probably leading to drastic and rapid modifications of the seafloor. In the following three years the breakwater was fully built, and the rate of system alteration slowed down. To fully understand whether this slower variation implies a return of the system to a different equilibrium, it will be necessary to plan new surveys over the same area and to compare the results with 3D hydrodynamic models including the effect of currents and waves. However, if the scouring will not stop, the new-built structures are probably going to face problems connected with their structural stability.
2. Dune fields drastically shrunk, going from an area of 437.9 · 10^3^ ± 0.1% *m*^2^ in 2011 to one of 212.5 · 10^3^ ± 0.1% *m*^2^ in 2016, with a reduction rate of 103916 *m*^2^/year in the first two-year-period. In the following three years the mean rate of disappearance decreased to 14927 *m*^2^/year. The reduction of dune fields corresponds to the reduction of the sand content in the seafloor sediment composition in favour of shell and shell detritus.

In view of global mean sea level rise and probable future construction of new “hard” defences against it, the combined use of very high resolution multibeam surveys and repeatable geomorphometric analysis proves to be an effective tool for knowledge-based coastal monitoring and management.

## Notations

*A* = surface of one grid cell

*B* = width of the breakwater

*d*_*max*_ = relative maximum scour depth

Δ*Z(i)* = difference of depth value for the grid cell i (i.e. the difference between the depth from survey *Z*_1_(*i*) and the survey *Z*_2_(*i*))

*h* = water depth

*H* = wave height

*i* = grid cell

*KC* = Keulegan-Carpenter number

λ = wavelength

*L* = width of protection layer (rip-rap) on the seafloor

*N* = total number of pixels contained in the morphological feature under consideration

*P* = perimeter of the morphological feature under investigation

*σ*_*area*_ = error in the area measurements

*σ*_*h*_ = horizontal TPU

*σ*_*V*_ = error in the volume measurements

*σ*_*z*_ = vertical TPU

*S* = area analysed

*U*_*c*_ = peak orbital velocity

*U*_*m*_ = depth-averaged current velocity

## Supporting information

S1 Table Grain size and textural parameters of sediment samples. The position of every sample is shown in Fig 5.
(PDF)

S2 Table Confusion matrix. (PDF)

S3 Table Main parameters describing every dune field in each year, with D1, D2 and D3 indicating the 2011, 2013 and 2016 datasets respectively. The position of every dune field is shown in S1 Fig.
(PDF)

S1 Fig Dune fields position. Areas (red polygons) and position of the dune fields in each year of the study. The properties of the dune fields are collected in S1 Table.
(TIF)

S2 Fig 2011 bathymetry. Hillshade of the 2011 bathymetry (raster resolution 0.5 m, 5 times vertical exaggeration). The colored polygons identify the different morphological features described in section 4 (detail of Fig 5d). Reprinted from Nautical Chart 226 under a CC BY license, with permission from Italian Hydrographic Institute, original copyright 2016.
(TIF)

S3 Fig 2013 bathymetry. Hillshade of the 2013 bathymetry (raster resolution 0.5 m, 5 times vertical exaggeration). The colored polygons identify the different morphological features described in section 4 (detail of Fig 5e). Reprinted from Nautical Chart 226 under a CC BY license, with permission from Italian Hydrographic Institute, original copyright 2016.
(TIF)

S4 Fig 2016 bathymetry. Hillshade of the 2016 bathymetry (raster resolution 0.5 m, 5 times vertical exaggeration). The colored polygons identify the different morphological features described in section 4 (detail of Fig 5f). Reprinted from Nautical Chart 226 under a CC BY license, with permission from Italian Hydrographic Institute, original copyright 2016.
(TIF)

S5 Fig Bathymetric difference between 2016 and 2011. a) 2011 morphological features and bathymetry; b) bathymetric difference between 2016 and 2011 and c) 2016 morphological features and bathymetry.
(TIF)

S6 Fig Comparison between backscatter, classified backscatter and seafloor image. Left column: backscatter represented in a grey scale image; central column) classified backscatter following the Jenks’ algorithm and right column) key seafloor image for every class of backscatter.
(TIF)

S7 Fig Water level and wave height in the study period, from 2011 to 2016. Top: water levels derived from the data of the Ispra gauge located in the southern jetty of Lido inlet. The red line indicates the water level of 140 cm above the medium sea level. The red stars highlight *Acqua alta* events higher than 140 cm; Bottom: wave height derived from the CNR platform Acqua Alta. The red line indicates a wave height of 3 m and the red stars highlight wave height higher than 3 m. In both figures, each survey is repersented between two parallel blue lines.
(TIF)
