# Supplementary material for: Tidal inlet seafloor changes induced by recently built hard structures
Source: PLoS One. 2019 Oct 16;14(10):e0223240. doi: 10.1371/journal.pone.0223240 (PMC6795416; doi:10.1371/journal.pone.0223240)
Supplement: S1 Table — The position of every sample is shown in Fig 5. (PDF) [file pone.0223240.s001.pdf]

Tidal inlet seafloor changes induced by recently built hard structures

Carlotta Toso[1,2*], Fantina Madricardo[1], Emanuela Molinaroli[2], Stefano Fogarin[1,2], Aleksandra Kruss[1], Antonio Petrizzo[1], Nicola Marco Pizzeghello[3], Luigi Sinapi[3] Fabio Trincardi[4],

1 Istituto di Scienze Marine-Consiglio Nazionale delle Ricerche, Arsenale - Tesa 104, Castello 2737/F, 30122 Venezia, Italy
2 Department of Environmental Sciences, Informatics and Statistics (DAIS), Università Ca' Foscari Venezia, Campus Scientifico, Via Torino 155, Mestre, VE, Italy
3 Istituto Idrografico della Marina, Passo all'Osservatorio 4, Genova 16134, Italy
4 Dipartimento Scienze del Sistema Terra e Tecnologie per l'Ambiente, Piazzale Aldo Moro 7, Roma, Italy

* toso.carlotta@gmail.com

Supporting Information

S1 Table Grain size and textural parameters of sediment samples. The position of every sample is shown in Fig 5.

Sample	Textural group	Mean	Sorting	Mode 1	Mode 2	D50	Gravel (%)	Sand (%)	Mud (%)
**A01**	Gravel	5685.66	6.49	12000.00	-	10869.65	83.3	12.9	3.8
**A02**	Sandy Mud	22.42	4.42	26.70	106.70	25.56	0.0	28.7	71.3
**A03**	Muddy Sandy Gravel	1082.41	7.33	302.00	12000.00	1200.40	45.8	48.5	5.6
**A04**	Gravelly Muddy Sand	227.34	4.11	213.50	-	222.39	8.4	82.3	9.3
**A05**	Muddy Sandy Gravel	958.34	8.63	302.00	12000.00	1395.53	47.1	44.8	8.1
**A06**	Muddy Sandy Gravel	510.80	14.10	213.50	12000.00	255.95	37.2	44.1	18.7
**A07**	Slightly Gravelly Sand	242.86	2.51	302.00	-	269.64	0.9	93.7	5.4
**A08**	Muddy Sandy Gravel	3422.55	11.67	47250.00	12000.00	7358.47	68.6	24.1	7.3
**A09**	Gravelly Sand	606.94	4.75	302.00	-	362.86	22.9	74.0	3.1
**A10**	Muddy Sandy Gravel	1439.15	6.58	6000.00	302.00	2542.22	55.5	39.8	4.7
**A11**	Gravelly Sand	378.84	3.28	302.00	-	338.15	8.3	87.6	4.1
**A12**	Gravelly Sand	688.69	4.61	427.00	12000.00	462.26	23.7	72.5	3.8
**A13**	Gravelly Sand	458.24	2.85	427.00	-	432.78	5.4	91.5	3.1
**A14**	Sandy Gravel	1180.10	7.75	6000.00	163.00	2296.56	52.3	43.8	3.9
**A15**	Sandy Gravel	1285.05	6.85	302.00	12000.00	1114.69	44.2	52.9	3.0
**A16**	Sandy Gravel	2308.41	5.69	12000.00	302.00	4044.50	60.8	37.4	1.8
**A17**	Sand	233.41	1.44	213.50	-	232.40	0.0	100.0	0.0
**A18**	Muddy Sandy Gravel	882.39	9.52	213.50	12000.00	346.88	39.8	53.1	7.1
**A19**	Muddy Sandy Gravel	849.83	8.60	213.50	6000.00	389.07	41.4	51.0	7.6
**A20**	Sandy Gravel	1408.69	6.53	427.00	1500.00	870.51	33.9	64.0	2.2
