# Supplementary material for: Tidal inlet seafloor changes induced by recently built hard structures
Source: PLoS One. 2019 Oct 16;14(10):e0223240. doi: 10.1371/journal.pone.0223240 (PMC6795416; doi:10.1371/journal.pone.0223240)
Supplement: S2 Table — (PDF) [file pone.0223240.s002.pdf]

Tidal inlet seafloor changes induced by recently built hard structures

Carlotta Toso[1,2*], Fantina Madricardo[1], Emanuela Molinaroli[2], Stefano Fogarin[1,2], Aleksandra Kruss[1], Antonio Petrizzo[1], Nicola Marco Pizzeghello[3], Luigi Sinapi[3] Fabio Trincardi[4],

1 Istituto di Scienze Marine-Consiglio Nazionale delle Ricerche, Arsenale - Tesa 104, Castello 2737/F, 30122 Venezia, Italy
2 Department of Environmental Sciences, Informatics and Statistics (DAIS), Università Ca' Foscari Venezia, Campus Scientifico, Via Torino 155, Mestre, VE, Italy
3 Istituto Idrografico della Marina, Passo all'Osservatorio 4, Genova 16134, Italy
4 Dipartimento Scienze del Sistema Terra e Tecnologie per l'Ambiente, Piazzale Aldo Moro 7, Roma, Italy

* toso.carlotta@gmail.com

Supporting Information

S2 Table Confusion matrix.

		**Samples Groups**				
		**I**	**II**	**III**	**total rows**	**users accuracy**
**Classified Backscatter**	**I**	1	1	0	2	0.50
	**II**	0	4	2	6	0.67
	**III**	0	2	10	12	0.83
	**total columns**	1	7	12	20	
	**producers accuracy**	1.00	0.57	0.83	**overall accuracy**	**0.75**
