# Supplementary material for: Tidal inlet seafloor changes induced by recently built hard structures
Source: PLoS One. 2019 Oct 16;14(10):e0223240. doi: 10.1371/journal.pone.0223240 (PMC6795416; doi:10.1371/journal.pone.0223240)
Supplement: S3 Table — The position of every dune field is shown in S1 Fig. (PDF) [file pone.0223240.s003.pdf]

Tidal inlet seafloor changes induced by recently built hard structures

Carlotta Toso[1,2*], Fantina Madricardo[1], Emanuela Molinaroli[2], Stefano Fogarin[1,2], Aleksandra Kruss[1], Antonio Petrizzo[1], Nicola Marco Pizzeghello[3], Luigi Sinapi[3] Fabio Trincardi[4],

1 Istituto di Scienze Marine-Consiglio Nazionale delle Ricerche, Arsenale - Tesa 104, Castello 2737/F, 30122 Venezia, Italy
2 Department of Environmental Sciences, Informatics and Statistics (DAIS), Università Ca' Foscari Venezia, Campus Scientifico, Via Torino 155, Mestre, VE, Italy
3 Istituto Idrografico della Marina, Passo all'Osservatorio 4, Genova 16134, Italy
4 Dipartimento Scienze del Sistema Terra e Tecnologie per l'Ambiente, Piazzale Aldo Moro 7, Roma, Italy

* toso.carlotta@gmail.com

Supporting Information

S3 Table Main parameters describing every dune field in each year, with D1, D2 and D3 indicating the 2011, 2013 and 2016 datasets respectively. The position of every dune field is shown in S1 Fig.

**Dune field**	λ **(m)**	**H (m)**	**Transversal Length (m)**	**Water depth (m)**	**Area** (m^2)
**D1_01**	10	0.25	100	11	33996
**D1_02**	10	0.3	70	10	3269
**D1_03**	6	0.15	50	11.6	3458
**D1_04**	5	0.15	90	11.8	9927
**D1_06**	10	0.3	50	11	12025
**D1_07**	15	0.4	80	7	6446
**D1_09**	6	0.15	130	6	14922
**D1_12**	7	0.2	55	6.5	29448
**D1_13**	4	0.2	140	8	92904
**D1_14**	5	0.15	50	13.6	6337
**D1_15**	5	0.4	220	13.4	107879
**D1_16**	10	0.4	70	13	12828
**D1_17**	4	0.1	55	15	17423
**D1_18**	3	0.15	50	13.5	25986
**D1_19**	4	0.15	17	12	6872
**D1_20**	7	0.2	45	12	4267
**D1_21**	10	0.2	110	12	17681
**D1_24**	9	0.15	35	9	6366
**D1_25**	8	0.2	140	11.5	21160
**D2_01**	10	0.5	80	12.5	14164
**D2_02**	10	0.15	210	11.9	22223
**D2_03**	20	0.2	100	6.5	7801
**D2_04**	8	0.2	220	7	85299
**D2_05**	6	0.3	200	13.8	124313
**D2_07**	8	0.1	40	12	2196
**D3_01**	10	0.2	160	7	52040
**D3_02**	5	0.2	100	12	54514
**D3_03**	10	0.2	180	11	68919
**D3_04**	10	0.6	65	12	11297
**D3_06**	7	0.15	100	7.5	25692
