# Supplementary figures and images for: Tidal inlet seafloor changes induced by recently built hard structures
Source: PLoS One. 2019 Oct 16;14(10):e0223240. doi: 10.1371/journal.pone.0223240 (PMC6795416; doi:10.1371/journal.pone.0223240)

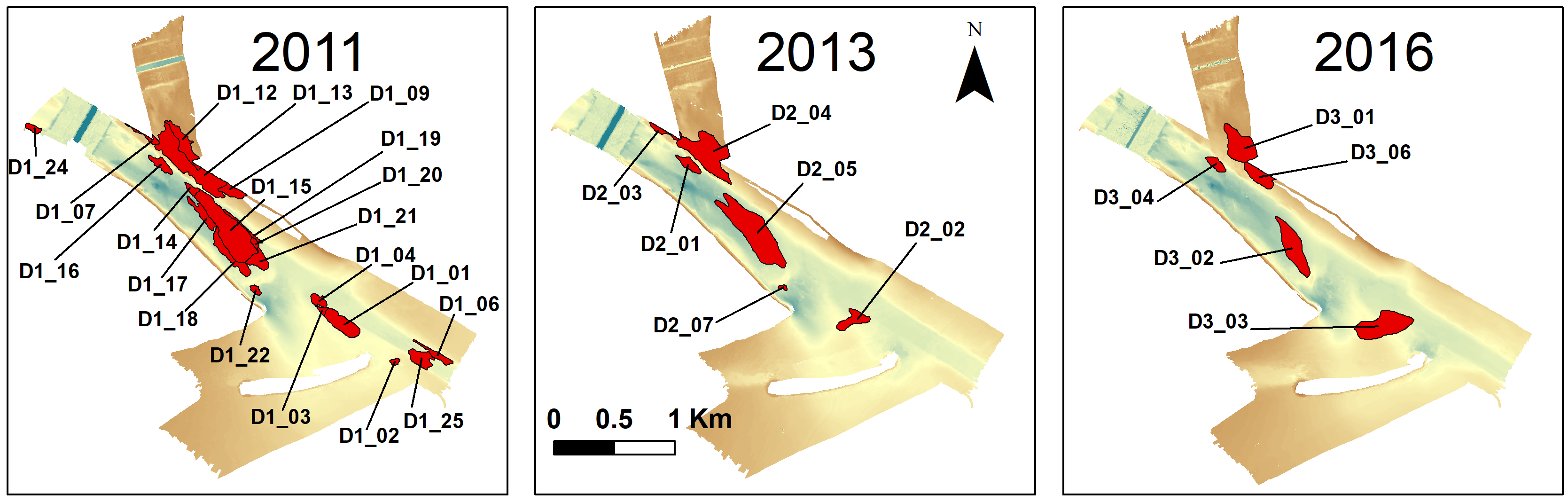

Supplement: S1 Fig — Areas (red polygons) and position of the dune fields in each year of the study. The properties of the dune fields are collected in S1 Table. (TIF) [file pone.0223240.s004.tif]

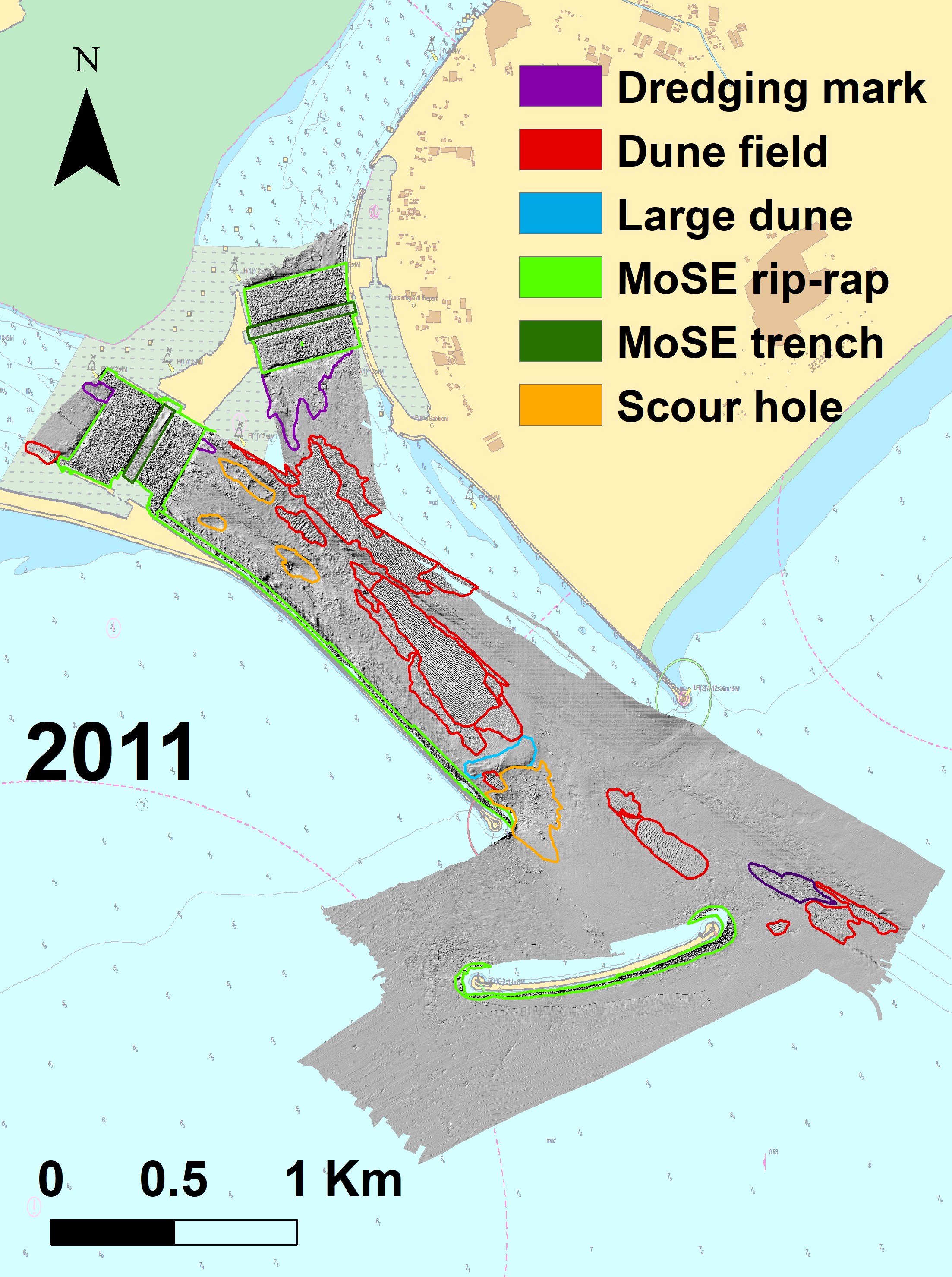

Supplement: S2 Fig — Hillshade of the 2011 bathymetry (raster resolution 0.5 m, 5 times vertical exaggeration). The colored polygons identify the different morphological features described in section 4 (detail of Fig 5d). Reprinted from Nautical Chart 226 under a CC BY license, with permission from Italian Hydrographic Institute, original copyright 2016. (TIF) [file pone.0223240.s005.tif]

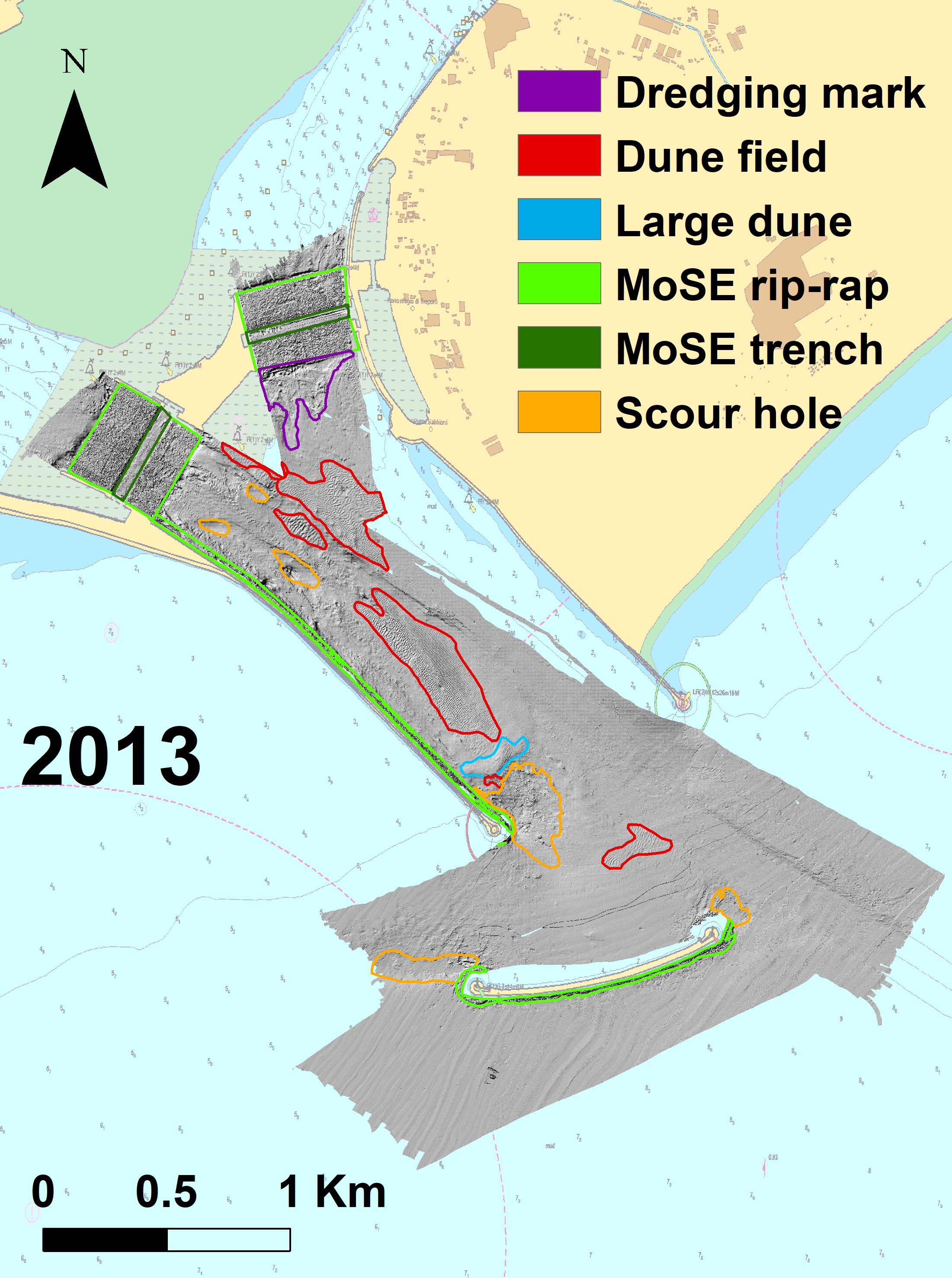

Supplement: S3 Fig — Hillshade of the 2013 bathymetry (raster resolution 0.5 m, 5 times vertical exaggeration). The colored polygons identify the different morphological features described in section 4 (detail of Fig 5e). Reprinted from Nautical Chart 226 under a CC BY license, with permission from Italian Hydrographic Institute, original copyright 2016. (TIF) [file pone.0223240.s006.tif]

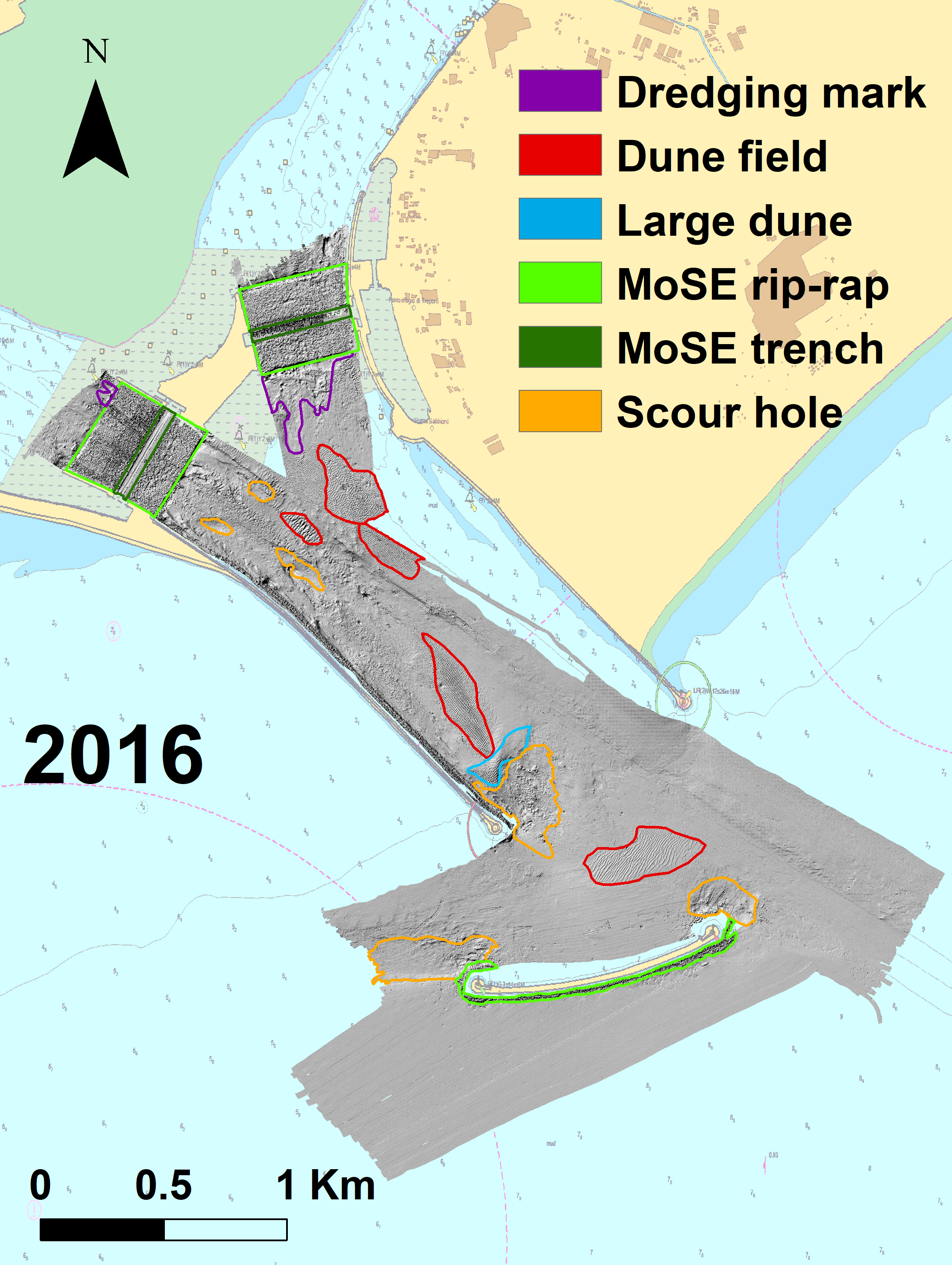

Supplement: S4 Fig — Hillshade of the 2016 bathymetry (raster resolution 0.5 m, 5 times vertical exaggeration). The colored polygons identify the different morphological features described in section 4 (detail of Fig 5f). Reprinted from Nautical Chart 226 under a CC BY license, with permission from Italian Hydrographic Institute, original copyright 2016. (TIF) [file pone.0223240.s007.tif]

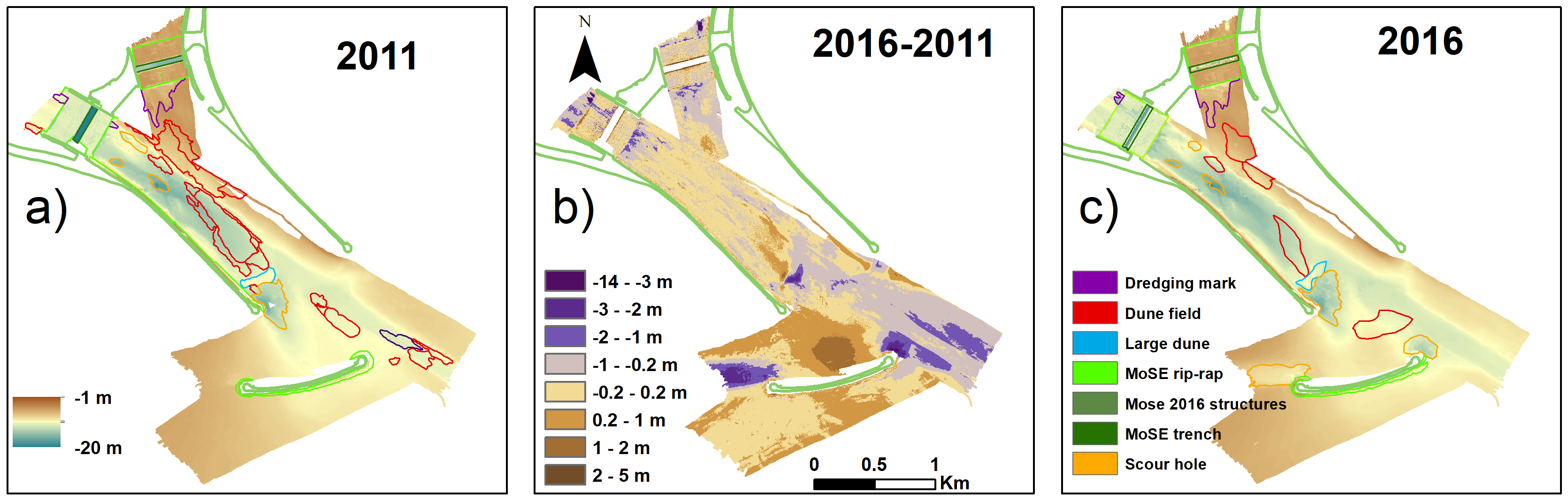

Supplement: S5 Fig — a) 2011 morphological features and bathymetry; b) bathymetric difference between 2016 and 2011 and c) 2016 morphological features and bathymetry. (TIF) [file pone.0223240.s008.tif]

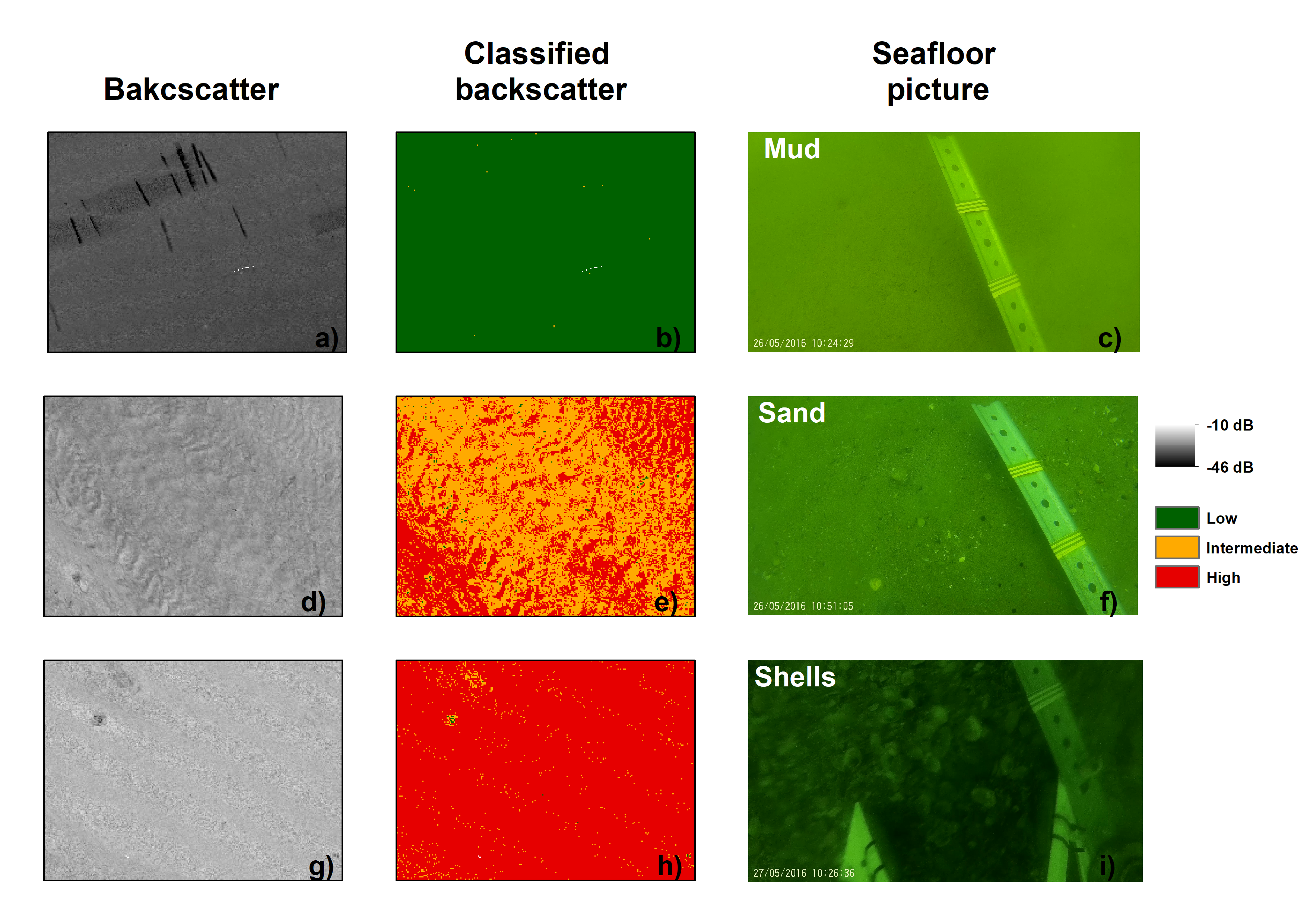

Supplement: S6 Fig — Left column: backscatter represented in a grey scale image; central column) classified backscatter following the Jenks’ algorithm and right column) key seafloor image for every class of backscatter. (TIF) [file pone.0223240.s009.tif]

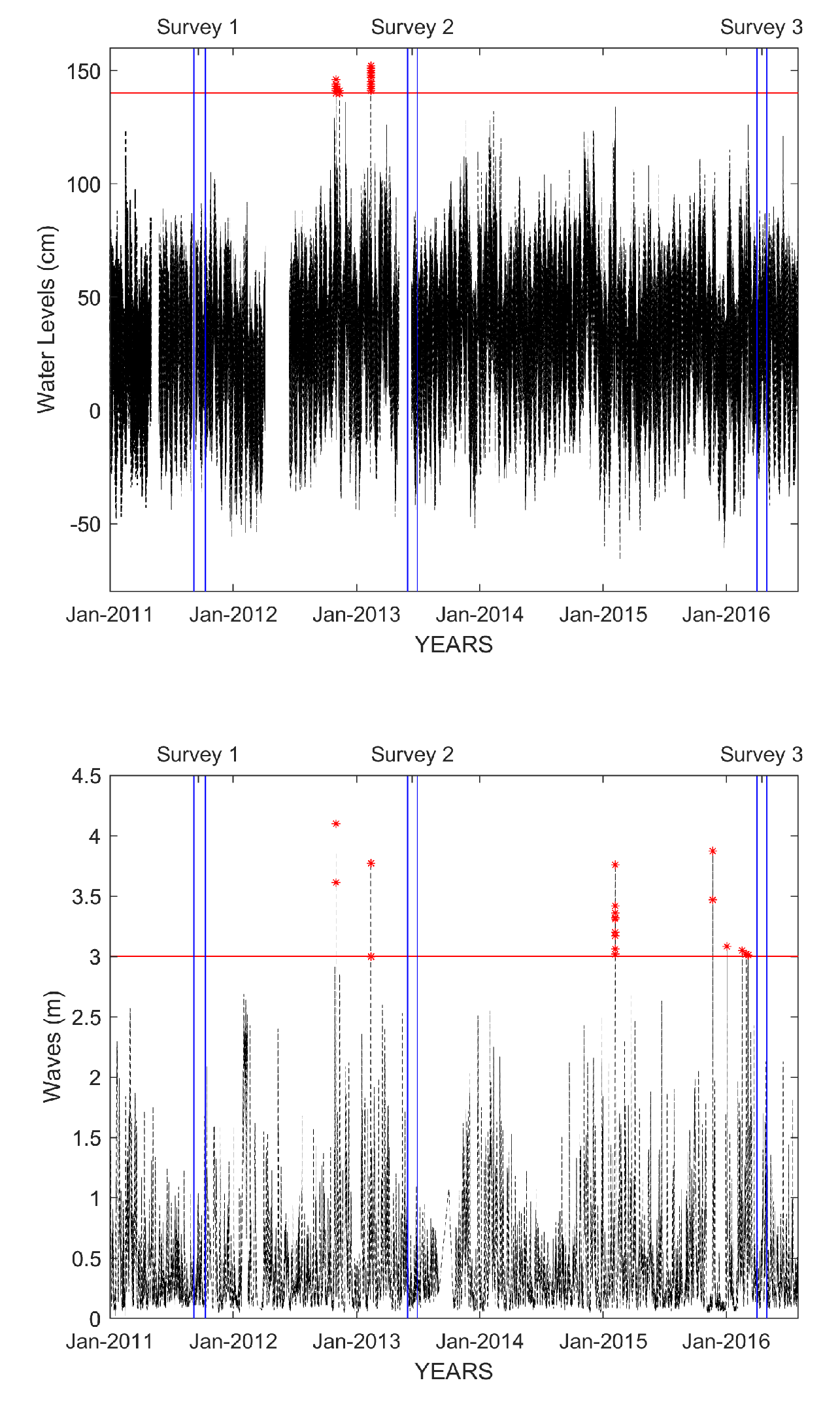

Supplement: S7 Fig — Top: water levels derived from the data of the Ispra gauge located in the southern jetty of Lido inlet. The red line indicates the water level of 140 cm above the medium sea level. The red stars highlight Acqua alta events higher than 140 cm; Bottom: wave height derived from the CNR platform Acqua Alta. The red line indicates a wave height of 3 m and the red stars highlight wave height higher than 3 m. In both figures, each survey is repersented between two parallel blue lines. (TIF) [file pone.0223240.s010.tif]
